# Top2a promotes the development of social behavior via PRC2 and H3K27me3

**DOI:** 10.1126/sciadv.abm7069

**Published:** 2022-11-23

**Authors:** Yijie Geng, Tejia Zhang, Ivy G. Alonzo, Sean C. Godar, Christopher Yates, Brock R. Pluimer, Devin L. Harrison, Anjali K. Nath, Jing-Ruey Joanna Yeh, Iain A. Drummond, Marco Bortolato, Randall T. Peterson

**Affiliations:** ^1^ Department of Pharmacology and Toxicology, College of Pharmacy, University of Utah, Salt Lake City, UT 84112, USA.; ^2^ The Graduate Program in Biophysical Sciences, The University of Chicago, Chicago, IL 60637, USA.; ^3^ Division of Cardiovascular Medicine, Beth Israel Deaconess Medical Center, Boston, MA 02115, USA.; ^4^ Cardiovascular Research Center, Department of Medicine, Massachusetts General Hospital, Harvard Medical School, Charlestown, MA 02129, USA.; ^5^ Metabolism Program, Broad Institute, Cambridge, MA 02142, USA.; ^6^ Davis Center for Aging and Regeneration, MDI Biological Laboratory, Bar Harbor, ME 04609, USA.

## Abstract

Little is understood about the embryonic development of sociality. We screened 1120 known drugs and found that embryonic inhibition of topoisomerase IIα (Top2a) resulted in lasting social deficits in zebrafish. In mice, prenatal Top2 inhibition caused defects in social interaction and communication, which are behaviors that relate to core symptoms of autism. Mutation of Top2a in zebrafish caused down-regulation of a set of genes highly enriched for genes associated with autism in humans. Both the Top2a-regulated and autism-associated gene sets have binding sites for polycomb repressive complex 2 (PRC2), a regulatory complex responsible for H3K27 trimethylation (H3K27me3). Moreover, both gene sets are highly enriched for H3K27me3. Inhibition of the PRC2 component Ezh2 rescued social deficits caused by Top2 inhibition. Therefore, Top2a is a key component of an evolutionarily conserved pathway that promotes the development of social behavior through PRC2 and H3K27me3.

## Teaser

A Top2a-PRC2-H3K27me3 pathway regulates expression of autism risk genes and drives development of sociality.

## INTRODUCTION

Sociality is broadly conserved across the animal kingdom, facilitating cooperation, reproduction, and protection from predation. Human infants exhibit an innate social preference behavior at birth: Infants less than an hour old fix their gaze on human face–like images longer than other images (*1*). Throughout life, this simple innate social drive serves as a foundation for the development of complex and versatile adult social behaviors. On the other hand, social dysfunction is a hallmark of several neurodevelopmental disorders (*2*) such as autism spectrum disorder. Deficits in early social behavior are linked to later diagnoses of autism (*3*). The onset of autism can be traced back to the prenatal stage (*4*–*6*), which coincides with the development of innate social behavior.

Despite its importance, little is known about the embryonic development of sociality. While many genes conferring a small amount of autism risk have been identified by genome-wide association studies (GWASs) and whole-exome sequencing (*7*–*9*), it is much less clear how they each contribute to autism etiology. Apart from these genetic factors, environmental factors are estimated to account for ~40% of autism risk (*10*, *11*), yet few have been identified. In this study, we investigated the development of sociality in zebrafish (*Danio rerio*) using a chemical genetic approach. The zebrafish has become an important model organism for behavioral studies because of its behavioral and neurophysiological similarities to humans. It has an evolutionarily conserved social brain network (*12*), enabling us to acquire insights into the social behavior development of potential relevance for mammals. Similar to that in humans, a robust social preference behavior emerges early in zebrafish (*13*, *14*). To restrict this investigation to developmental events occurring during the embryonic stage, we conducted a chemical screen that allows us to precisely apply and withdraw chemicals to and from zebrafish embryos during embryonic development. We screened an annotated library containing chemicals with known biological targets to help rapidly identify downstream biological targets.

Using a high-throughput behavioral analysis system that we named Fishbook, we screened 1120 known drugs and found that the embryonic inhibition of topoisomerase IIα (Top2a) resulted in social deficits in juvenile zebrafish. In mice, prenatal Top2 inhibition caused postnatal behavioral defects that are specifically related to the two core symptoms of autism: social communication impairments and increased perseveration. Several known environmental risk factors of autism are Top2 inhibitors (*15*–*17*), indicating that Top2 inhibition may be a common mechanism through which environmental insults contribute to autism risk.

To elucidate the Top2-mediated mechanism of action, we performed RNA sequencing (RNA-seq) of Top2a mutant zebrafish and observed the down-regulation of a set of genes highly enriched for autism risk genes. Using a custom analysis pipeline, we found that both the Top2a-dependent gene set and a set of autism-associated genes maintained by the Simons Foundation for Autism Research (SFARI genes hereafter) are selectively targeted by polycomb repressive complex 2 (PRC2), a regulatory complex responsible for depositing H3K27 trimethylation (H3K27me3). In corroboration of this finding, we found both gene sets to be highly enriched for H3K27me3. Notably, chemical inhibition of the PRC2 component Ezh2 rescued social deficits caused by Top2 inhibition in zebrafish, indicating that Top2a likely functions by antagonizing PRC2/H3K27me3-mediated gene silencing. These findings identify Top2a as a key component of an evolutionarily conserved pathway that promotes the development of social behavior through PRC2 and H3K27me3.

## RESULTS

### Fishbook: A scalable social behavior assay system

**Fig. 1.**
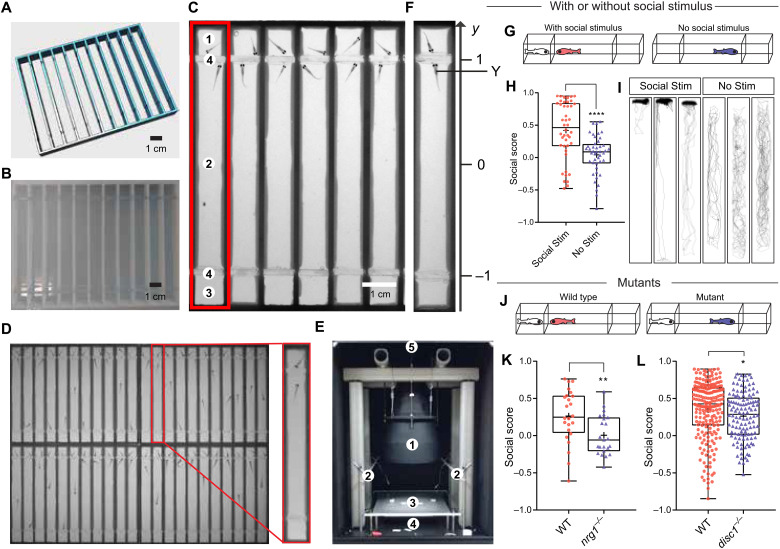
The Fishbook assay. (**A** and **B**) The design and a 3D printed array of Fishbook arenas. (**C**) Screenshot of a Fishbook assay. Red rectangle: Fishbook arena; 1: social stimulus compartment; 2: social test compartment; 3: empty control compartment; 4: transparent windows. (**D**) An array of 44 arenas. (**E**) Fishbook imaging station. 1: telecentric lens; 2: white LED panels providing ambient light; 3: sample deck; 4: 850-nm infrared LED backlight panel; 5: charge-coupled device camera. (**F**) Social score is calculated as the average *y*-axis position for the duration of a test. (**G** to **I**) Fishbook with or without social stimulus fish (G), showing boxplots (H) and representative tracking plots (I) of WT fish tested with social stimulus (Social Stim; *n* = 44) or without social stimulus (No Stim; *n* = 44). In each boxplot, the box encloses data points from the 25th to 75th percentile, the horizontal line and cross mark the median and the mean, and the lines above and below the box reach data points with the maximum and minimum values. ****P < 0.0001. (**J** to **L**) Fishbook detects social deficits in mutants (J), comparing WT (*n* = 24) and homozygous *nrg1*-knockout (*nrg1^−/−^*; *n* = 23) fish, both from a heterozygous (*nrg1^+/−^*) incross and genotyped individually after Fishbook assay (K), and WT sibling (*n* = 209) and homozygous *disc1*-knockout (*disc1^−/−^*; *n* = 122) fish (L). *P < 0.05 and **P < 0.01.

Zebrafish develop a robust social preference for age-matched conspecifics at 3 weeks of age (*13*). To quantitatively assess this behavior, we developed a scalable and fully automated assay system named Fishbook. Briefly, a test arena (Fig. 1A) was manufactured by three-dimensional (3D) printing (Fig. 1B). Each arena (Fig. 1C, red rectangle) is a long rectangular lane printed using nontransparent material, divided into three parts by two transparent windows (4): The longer middle part serves as the test compartment (2), one of the two smaller end-compartments contains a live fish as social stimulus (1), and the other end-compartment is left empty (3) (movie S1). To maximize throughput, we grouped 44 arenas together (Fig. 1D) and constructed a unique telecentric lens–based imaging system that enables us to simultaneously image all 44 arenas while avoiding optical obstruction by the nontransparent arena walls (Fig. 1E). Each person can screen >1000 fish per day using a single Fishbook apparatus.

A social score with values ranging between −1 and 1 was defined to quantitatively measure social behavior (Fig. 1F). Wild-type (WT) fish typically spend a substantial amount of time near the social stimulus fish, generating high social scores; without a social stimulus, test subjects swim randomly, giving rise to an average social score close to 0 (Fig. 1, G to I). Consistent with a previous report (*13*), this preference is lost in the dark (fig. S1A), indicating that it is driven primarily by visual cues. Social score is also drastically reduced when the social stimulus fish is replaced by a pebble of similar size (fig. S1B), suggesting that this preference behavior is highly specific to social cues and cannot be explained solely by curiosity toward an unfamilliar object and is in agreement with the previous finding that movement and swim bout structures are what induce social interaction in zebrafish (*18*). To validate its power in recognizing social deficits, we examined fish having loss-of-function mutations of genes linked to autism and schizophrenia, including *Nrg1* (Fig. 1K and fig. S1C) and *Disc1* (Fig. 1L and fig. S1D). Social scores are significantly reduced in both mutants, demonstrating that the Fishbook assay can effectively detect social defects caused by a single genetic mutation.

### A systematic screen identifies fluoroquinolones as inhibitors of social development

**Fig. 2.**
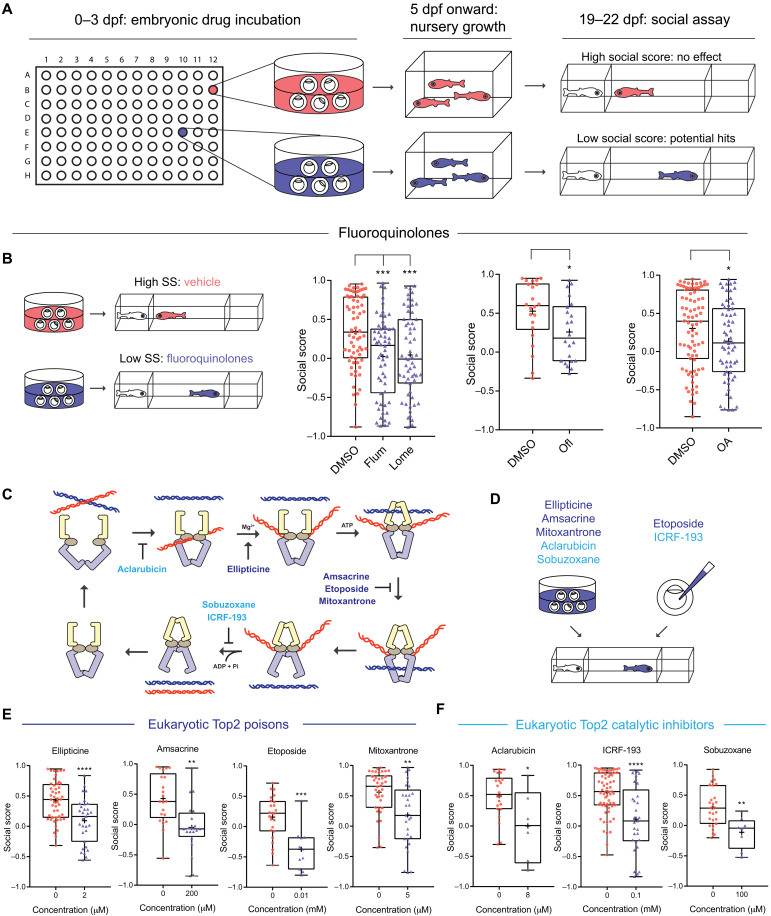
Screening for inhibitors of social development identifies fluoroquinolones and other Top2 inhibitors. (**A**) Screening workflow. (**B**) Fluoroquinolones induce social deficits. Comparing DMSO (*n* = 68, 22, and 82, from left to right) with flumequine (Flum; 25 μM; *n* = 56), lomefloxacin (Lome; 100 μM; *n* = 60), ofloxacin (Ofl; 200 μM; *n* = 22), and oxolinic acid (OA; 200 μM; *n* = 62). SS, sodium salicylate. (**C**) The catalytic cycle of Top2 and inhibitors targeting different steps of the cycle. ATP, adenosine 5′-triphosphate; ADP, adenosine 5′-diphosphate. (**D** to **F**) Eukaryotic Top2 inhibitors induce social deficits (D). Comparing fish treated with vehicle control (0 μM in each panel); Top2 poisons ellipticine (0 μM: *n* = 48; 2 μM: *n* = 31), amsacrine (0 μM: *n* = 21; 200 μM: *n* = 20), etoposide (0 μM: *n* = 24; 10 μM: *n* = 11), and mitoxantrone (0 μM: *n* = 38; 5 μM: *n* = 29) (E); and Top2 catalytic inhibitors aclarubicin (0 μM: *n* = 22; 8 μM: *n* = 6), ICRF-193 (0 μM: *n* = 65; 100 μM: *n* = 29), and sobuzoxane (0 μM: *n* = 27; 100 μM: *n* = 8) (F). *P < 0.05, **P < 0.01, ***P < 0.001, and ****P < 0.0001.

Using Fishbook, we screened the Prestwick library, which contains 1120 compounds with known biological targets. Briefly, embryos were exposed to chemicals during the first 3 days of development [0 to 3 days postfertilization (dpf)], raised to 3 weeks of age, and tested in the Fishbook assay (Fig. 2A). Hits were defined as having an average social score < 0.1. Because of the relatively large variation expected for a behavior-based assay (*19*), to reduce the number of false-positive hits, primary screening was performed in duplicates, and hits were confirmed by repeat testing. Four confirmed hits were found, including flumequine, lomefloxacin, ofloxacin, and oxolinic acid (Fig. 2B and fig. S2A). These four compounds were the only validated hits from the screen, and all belonged to the same class of antibiotics named fluoroquinolones. A previous publication reported modulation of zebrafish shoaling behavior by long-term exposure to a mixture of three fluoroquinolones (ofloxacin, ciprofloxacin, and enrofloxacin) and three tetracyclines, suggesting a potential link between fluoroquinolones and zebrafish social behavior, which is in line with our observation, but did not examine the effect of fluoroquinolones alone without tetracyclines or provide insights on their molecular mechanisms (*20*).

### Top2 inhibition is responsible for the social deficit phenotype

Although fluoroquinolones are best known as antibiotics targeting the prokaryotic type II topoisomerases, they also inhibit the eukaryotic Top2, albeit at higher doses. For example, the median inhibitory concentrations of ofloxacin and lomefloxacin against HeLa cell Top2 are 365 and 613 μM (*21*), and their effective doses in the Fishbook assay are 200 and 100 μM, respectively. Prior studies have also suggested a link between Top2 and autism (see Discussion for details) (*22*–*26*). We thus hypothesized that fluoroquinolones inhibit the development of sociality by targeting zebrafish Top2.

To test this hypothesis, we examined several structurally diverse eukaryotic Top2 inhibitors (fig. S2B). They target different steps of the Top2 catalytic cycle (Fig. 2C) (*27*, *28*) and can be grouped into two categories based on their mechanisms of action, including Top2 poisons (ellipticine, amsacrine, etoposide, and mitoxantrone) and Top2 catalytic inhibitors (aclarubicin, ICRF-193, and sobuzoxane). Through embryonic exposure (Fig. 2D), all seven structurally diverse Top2 poisons and inhibitors induced social deficits (Fig. 2, E and F). We also tested several environmental chemicals that were known to inhibit eukaryotic Top2 activity, including chlorpyrifos, chlorpyrifos oxon, and genistein (see Discussion for more information), and observed similar social deficits (fig. S2C). It is worth noting that, under the doses specified in the figure legends (Figs. 2, B, E, and F, and 3C, and fig. S2C), all Top2 inhibitor–treated fish can feed normally and actively avoid capture, indicating that their vision is not substantially impaired. An optomotor response assay (*29*) showed no visual defects in 21 dpf fish that received embryonic treatment of flumequine and sodium salicylate, a Top2a-selective inhibitor (see more in the upcoming sections; fig. S2D). We also did not observe gross morphological defects or other apparent abnormal behaviors in fish treated by Top2 inhibitors, when applied at the same doses that induced social deficits (Figs. 2, B, E, and F, and 3C, and fig. S2C). These results demonstrated that inhibition of zebrafish Top2 can indeed induce social deficits.

**Fig. 3.**
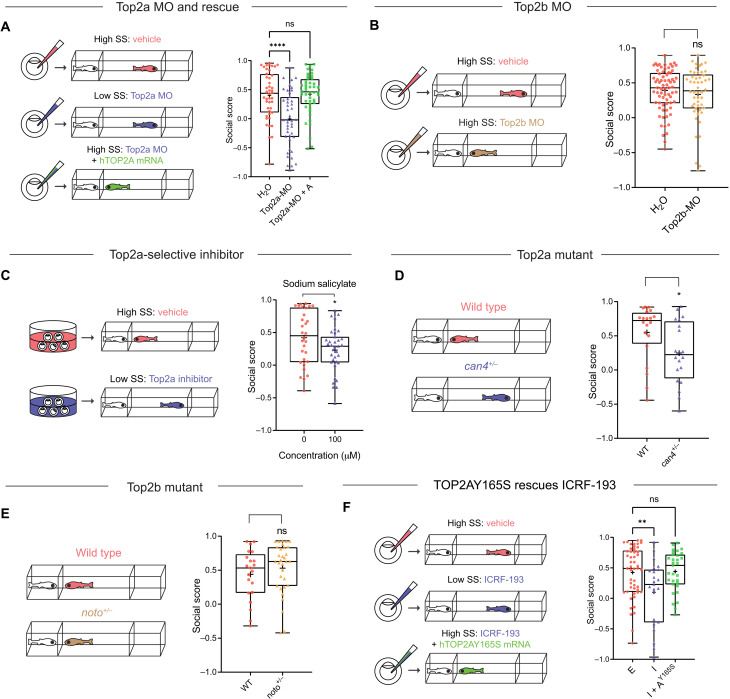
Top2a, rather than Top2b, is required for the development of social behavior. (**A**) Top2a-MO (0.05 mM; *n* = 44) induces social deficits compared to vehicle (H_2_O, *n* = 44), which is rescued by *hTOP2A* mRNA (250 ng/μl; Top2a-MO + A, *n* = 44). (**B**) Top2b-MO (0.05 mM; *n* = 50) does not induce social deficits compared to vehicle (H_2_O, *n* = 71). (**C**) Top2a-selective inhibitor sodium salicylate (100 μM) induces social deficits. (**D**) Heterozygous *can4* mutant (*can4^+/−^*, *n* = 20) exhibits social deficits compared to WT (*n* = 20). Both mutant and WT were acquired from a heterozygous (*can4^+/−^*) incross and genotyped individually after Fishbook assay. (**E**) Heterozygous *noto* mutant (*noto^+/−^*, *n* = 32) shows no social deficits compared to WT (*n* = 21). Both mutant and WT were acquired from a heterozygous (*noto^+/−^*) incross and genotyped individually after Fishbook assay. (**F**) ICRF-193 (I; 0.1 mM; *n* = 23) induced social deficits compared to the vehicle control ethanol (E; 1%; *n* = 50), which is rescued by *hTOP2AY165S* mRNA (250 ng/μl; I + A^Y165S^, *n* = 31). ns, not significant; *P < 0.05, **P < 0.01, and ****P < 0.0001.

### Top2 inhibition is unlikely to function by inhibiting transcription or genomic stability

Apart from its role in facilitating replication, Top2 plays important parts in promoting transcription and genomic stability (*30*). We therefore investigated whether the effect of Top2 inhibition can be attributed to deficits in one of these two apparent mechanisms. We first examined two RNA polymerase II inhibitors, actinomycin D and triptolide, but did not observe social deficits (fig. S2, E and F). We then tested two DNA double-strand break (DSB) inducers, bleomycin and hydroxyurea, and again found that neither compound induced social deficits (fig. S2G). This result is also supported by the fact that while Top2 poisons induce DSBs, catalytic inhibitors do not, yet both classes of Top2 inhibitors cause social deficits (Fig. 2, E and F). Together, these data suggest that Top2 inhibition does not function through direct inhibition of general transcription or DNA instability.

### Top2a, but not Top2b, is required for the development of social behavior

We next investigated which isoform, Top2a or Top2b, was responsible for the social deficit phenotype. We first applied two splice-blocking morpholino oligonucleotides (MOs) (fig. S3A). Compared to permanent genomic editing methods such as CRISPR, MO’s transient-acting nature better mimics the effects of the Top2 inhibitors. Note that maternal Top2a mRNAs will escape inhibition by Top2a-MO: They are present in the embryos before maternal-to-zygotic transition (MZT), which occurs at ~3 hours after fertilization; their presence is reduced after MZT but is still maintained at a level that is detectable by real-time polymerase chain reaction (PCR) up to 3 dpf (*31*). Following injection, Top2a-MO induced social deficits, which were rescued by coinjecting *hTOP2A* mRNA (Fig. 3A and fig. S3B). Top2b-MO, however, failed to induce social deficits (Fig. 3B). The Top2a-selective inhibitor sodium salicylate also induced social deficits (Fig. 3C) when applied at a dose known to confer its isoform selectivity (*28*).

We then examined two loss-of-function mutants of Top2a [*can4* (*32*)] and Top2b [*noto* (*33*)]. Because homozygous mutants are lethal, we established breeding colonies using heterozygous (het) mutants. Incrossing het mutants generated mixed populations composed of ~^1^/_3_ WT and ^2^/_3_ het offspring. They were assayed blind in Fishbook, i.e., with no prior knowledge of each fish’s genetic background, and genotyped individually. Het Top2a mutant (*can4^+/−^*) exhibited reduced social score compared to WT sibling (Fig. 3D), while het Top2b mutant (*noto^+/−^*) did not show reduction in social score (Fig. 3E).

Last, we tested whether transient overexpression of Top2a can rescue the Top2 inhibitor–induced social deficit. A potential concern is that overexpressed WT hTOP2A protein may simply bind to and “soak up” a Top2 inhibitor, leaving less free compound to interact with its real target, resulting in false-positive rescue. We therefore generated a hTOP2A mutant (Y165S) that is functionally active but resistant to ICRF-193 (*34*). Transient overexpression of hTOP2AY165S by mRNA injection successfully rescued ICRF-193 (Fig. 3F and fig. S3C), demonstrating that Top2a alone can rescue Top2 inhibition. Together, these results suggest that Top2a is the isoform responsible for the social deficit phenotype.

### Prenatal inhibition of Top2 induces social deficits and other autism-related behavioral defects in mice

**Fig. 4.**
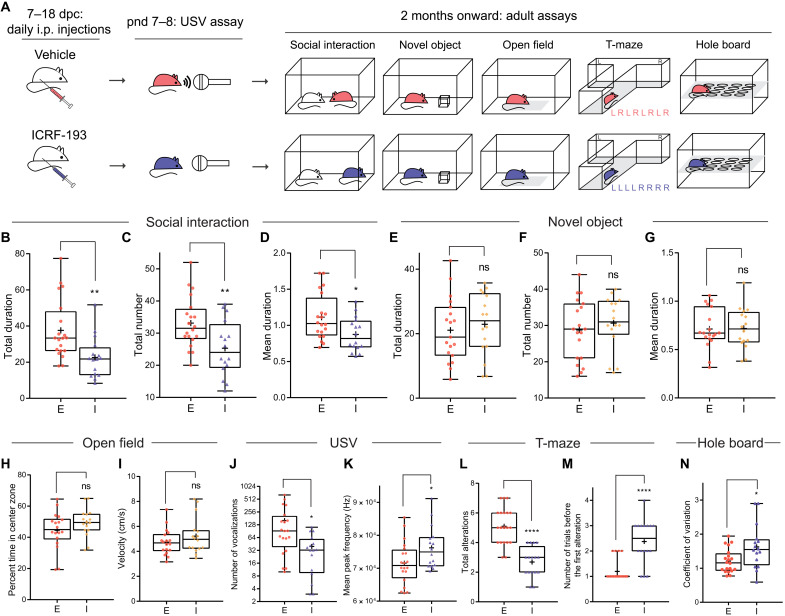
Prenatal inhibition of Top2 induces social communication impairments and increased perseveration in mouse. (**A**) Experimental design for prenatal ICRF-193 injection and subsequent experiments for the offspring. L, left; R, right. (**B** to **D**) ICRF-193 (I, *n* = 16) reduces the total duration (B), total number (C), and mean duration (D) of social investigation compared to vehicle control ethanol (E, *n* = 20). (**E** to **G**) ICRF-193 (*n* = 16) does not reduce the total duration (E), total number (F), and mean duration (G) of novel object investigation compared to ethanol (*n* = 19). (**H** and **I**) ICRF-193 (*n* = 16) does not change the percentage of time spent in the center zone (H) and velocity (I) of mice in the open-field assay compared to ethanol (*n* = 18). (**J** and **K**) ICRF-193 (*n* = 16) reduces the total number (J) but increases the mean peak frequency (K) of USV compared to ethanol (*n* = 20). (**L** and **M**) ICRF-193 (*n* = 16) reduces the total number of alterations (the number of times a mouse enters an alternative arm compared to the previous trial) (L) while increasing the number of trials before the first alteration (M) compared to ethanol (*n* = 20) during a T-maze assay that consisted of eight trials. (**N**) ICRF-193 (*n* = 16) increases the coefficient of variation in the hole-board assay compared to ethanol (*n* = 20). *P < 0.05, **P < 0.01, and ****P < 0.0001.

To determine whether this Top2-mediated mechanism is conserved in mammals, we assessed the effect of prenatal Top2 inhibition in mice. Pregnant mice received daily intraperitoneal injections of ICRF-193 or vehicle control from 7 to 18 days post coitum (dpc). Offspring grew to adult stage without observable differences in gross morphology or body weight (fig. S3D) compared to vehicle control or no-treatment groups. Behavioral assays were conducted 2 to 10 months after birth (Fig. 4A).

We first conducted a reciprocal social interaction assay (*35*), in which two sex- and age-matched mice, including a test subject and a social stimulus mouse, were placed into an unfamilliar cage and video recorded. The number and duration of interactions between a test subject and the control mouse were manually scored by a blinded reviewer. ICRF-193–treated mice showed significantly reduced total duration, total number, and mean duration (Fig. 4, B to D) of social investigations compared to the vehicle control. We then performed a novel object exploration assay to determine whether the ICRF-193 mice lack interest in or even avoid novel stimuli in general. ICRF-193 mice did not show significantly altered object exploration compared to controls, as quantified by the total duration, total number, and mean duration (Fig. 4, E to G) of their interactions with the novel object (a wooden cube). We also observed no significant changes in motion patterns in an open-field assay, as measured by the time spent in the center of the test arena and average velocity (Fig. 4, H and I), indicating intact motor capabilities and normal stress responses for the ICRF-193 mice. These results indicate that social behaviors are selectively targeted by prenatal Top2 inhibition, while exploratory behaviors, motor activity, and stress response remain intact.

We then investigated other autism-related behavioral deficits including impaired communication and perseveration. We examined ultrasonic vocalization (USV) of the pups for potential social communication deficits. When briefly separated from their mothers, ICRF-193–treated pups exhibited significantly reduced numbers of USVs compared to controls (Fig. 4J), indicating a profound impairment in their communication. The ICRF-193–treated pups also showed a significantly elevated mean peak frequency in their vocalizations (Fig. 4K), a phenotype that was also found in established mouse models of autism (*36*, *37*). We then examined restrictive and repetitive behaviors using T-maze and hole-board assays (*35*). ICRF-193 mice repeatedly explored the same arm of a T-maze, as shown by their significantly reduced number of alternative explorations and delayed onset of first alteration (Fig. 4, L and M) compared to the controls. Hole-board assay also revealed a significantly elevated coefficient of exploratory variation, a measure of repetitive tendencies (*35*), in ICRF-193 mice (Fig. 4N). These results demonstrate that prenatal inhibition of Top2 induces behavioral deficits related to the core symptoms of autism in mice.

### Top2a depletion selectively down-regulates autism risk genes

**Fig. 5.**
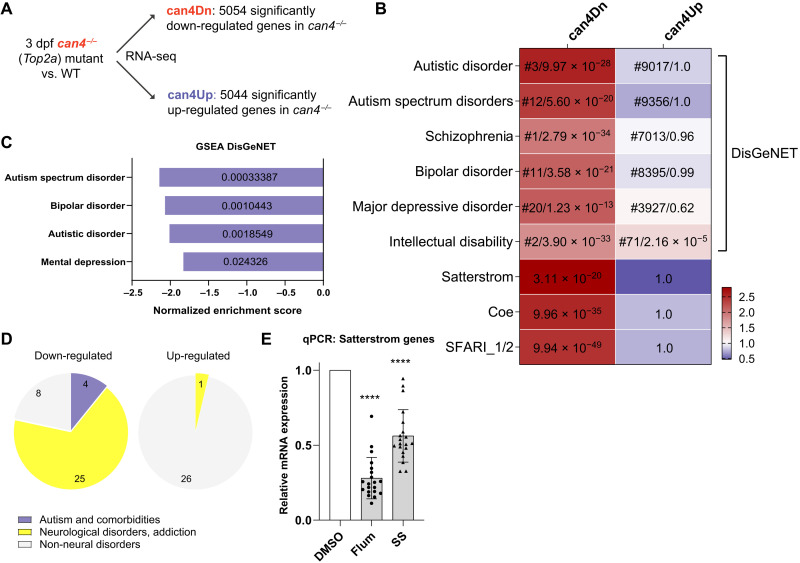
RNA-seq reveals down-regulation of autism risk genes caused by Top2a depletion. (**A**) RNA-seq experimental design. (**B**) can4Dn, but not can4Up, is selectively enriched for autism and its comorbidities risk genes. For each cell, color represents odds ratio. Numbers in the top six rows represent ranking out of 24,166 diseases in the DisGeNET library (before slash) and adjusted *P* value (after slash). Numbers in the bottom three rows represent adjusted *P* value. SFARI_1/2, high-confidence SFARI genes with a gene score of 1 or 2. (**C** and **D**) GSEA analysis using the DisGeNET library shows enrichment for autism and its comorbidities risk genes (C) and neurological condition risk genes (D) in down-regulated. Value inside each bar represents false discovery rate (FDR) (C). Significance: FDR < 5%. (**E**) qPCR shows the down-regulation of Satterstrom genes in the brain of adult zebrafish that received embryonic treatment of 15 μM flumequine or 100 μM sodium salicylate compared to DMSO control. ****P < 0.0001.

On the basis of the observed link between Top2a inhibition and autism-related behavioral deficits, we investigated whether Top2a depletion can selectively modulate the expression of autism risk genes using RNA-seq of 3 dpf *can4^−/−^* (Top2a) mutants (Fig. 5A). Because of the presence of maternal Top2a mRNA up to 3 days (although at low levels after MZT) of embryogenesis (*31*), *can4^−/−^* embryos are expected to be partially depleted of Top2a during the first few days of embryonic development. We found 8634 significantly (adjusted *P* < 0.05) up-regulated genes and 8589 significantly down-regulated genes in *can4^−/−^* mutants compared to WT controls. Top2a was down-regulated as expected (adjusted *P* = 2.16 × 10^−73^). We used the human orthologs of zebrafish genes for subsequent analyses, which include 5044 human orthologs for the down-regulated genes (hereafter referred to as *can4^−/−^* down-regulated genes or can4Dn) and 5054 human orthologs for the up-regulated genes (*can4^−/−^* up-regulated genes or can4Up; data S1).

To identify diseases that most closely associate with genes affected by Top2a depletion, we analyzed the two disease-gene libraries DisGeNET (*38*) and GLAD4U (*39*) using overrepresentation analysis (ORA) to search for disease risk gene sets that are enriched (overrepresented) in the can4Dn and can4Up gene lists. can4Dn is enriched for genes associated with autism and its comorbid conditions including schizophrenia, bipolar disorder, depression, and intellectual disability; these diseases consistently rank at the top of the list among the 24,166 diseases in the DisGeNET library and the 3071 in GLAD4U, demonstrating a high degree of specificity for these diseases (Fig. 5B and fig. S4A). can4Up is not enriched for most of these diseases except modestly for intellectual disability in DisGeNET. We also found a similar pattern of enrichment in independently curated disease gene sets related to autism and its comorbidities [Satterstrom (*8*), Coe (*9*), SFARI (*40*, *41*), AutismKB 2.0 (*42*), DisGeNET (*38*), GLAD4U (*39*), BDgene (*43*), PsyGeNET (*44*), SZGene (*45*), SysID (*46*), and GEPAD (Genomics England’s PanelApp database) (*47*)] (Fig. 5B and fig. S4B). Using gene set enrichment analysis (GSEA) as an orthogonal approach, we again found significant enrichment (5% false discovery rate) of autism and its comorbidities risk genes only in the down-regulated genes (Fig. 5, C and D, and figs. S4C and S5).

The can4 homozygous mutant displays morphological defects and potential loss of neuronal population during development (*31*), which may skew the RNA-seq result. To examine whether the major autism genes are similarly down-regulated in Top2 inhibitor–treated fish and whether this change in gene expression is persistent after removal of drugs, we conducted quantitative PCR (qPCR) analysis in drug-treated WT fish. Using adult zebrafish (2-month-old) brain tissue, we found that the embryonic treatment of flumequine and sodium salicylate induced persistent down-regulation of the 13 highest-ranking genes reported by Satterstrom *et al.* (*8*) (Satterstrom genes; Fig. 5E and table S2), indicating that Top2 inhibitor treatments also induced down-regulation of key autism risk genes, similar to what was found in the can4 homozygous mutant, and that this change in gene expression persisted to the adult stage.

Pathway [Kyoto Encyclopedia of Genes and Genomes (KEGG) and REACTOME] and Gene Ontology (GO) ORA analyses also revealed enrichment of genes related to pathways that are believed to contribute to autism etiology (*48*), including axon guidance, neuronal development, glutamatergic signaling, and synaptic transmission, in can4Dn but not can4Up (fig. S6A). GSEA analysis using these libraries showed similar enrichments only in the down-regulated genes (fig. S6B).

To eliminate any potential bias in our enrichment analyses caused by naturally occurring enrichment or depletion of autism-associated genes in zebrafish, we examined the specificity of our analyses using a permutation assay. Because can4Dn and can4Up both contain close to 5000 genes, we randomly selected 1000 lists of 5000 genes of the 14,989 human genes with zebrafish orthologs and repeated the ORA analyses for these gene sets to create a null distribution. We found that can4Dn, but not can4Up, consistently gives significantly higher odds ratios compared to the null distribution, thus validating the specificity of the observed enrichment of autism risk genes (figs. S7 to S9).

### can4Dn and autism risk genes share similar upstream regulators

**Fig. 6.**
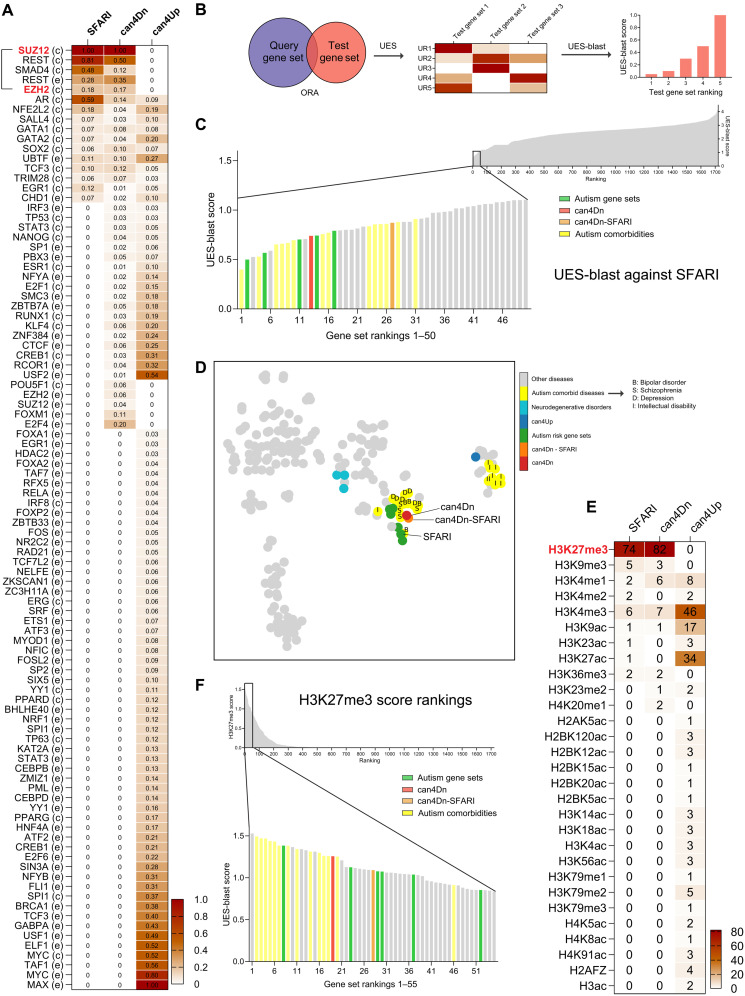
PRC2 and H3K27me3 are common upstream regulators of can4Dn and autism risk genes. (**A**) SFARI genes and can4Dn, but not can4Up, share very similar enrichment profiles for upstream regulators. Value in each cell represents the significance score (Materials and Methods). In row labels, c indicates ChEA, and e indicates ENCODE, indicating the source of ChIP-seq data. (**B**) UES analysis and UES-blast workflow. ORA analysis finds significantly enriched upstream regulators for a test gene set using reference gene sets from the ChIP-X library. A significance score is calculated for each upstream regulator (Materials and Methods), creating a signature for each test gene set, which we named UES. A blast-style querying algorithm (UES-blast) then identifies test gene sets with the most similar UES compared to a query gene set, e.g., SFARI genes. UR, upstream regulator. (**C**) UES-blast (Materials and Methods) shows that can4Dn, can4Dn-SFARI, and several independently curated autism risk gene sets rank high among control gene sets against the query gene set of SFARI genes, indicating that they share a very similar UES with the SFARI genes. (**D**) tSNE clustering of can4Dn, can4Dn-SFARI, autism risk gene sets, and DisGeNET gene sets (only those containing >500 genes). Letters label autism comorbidities risk gene sets as follows: I, intellectual disability; D, depression; S, schizophrenia; B, bipolar disorder. (**E**) Genes in the SFARI and can4Dn gene sets, but not in can4Up, are specifically enriched for the H3K27me3 mark. (**F**) can4Dn, can4Dn-SFARI, and autism risk gene sets have the highest H3K27me3 scores when compared to the control gene sets.

**Fig. 7.**
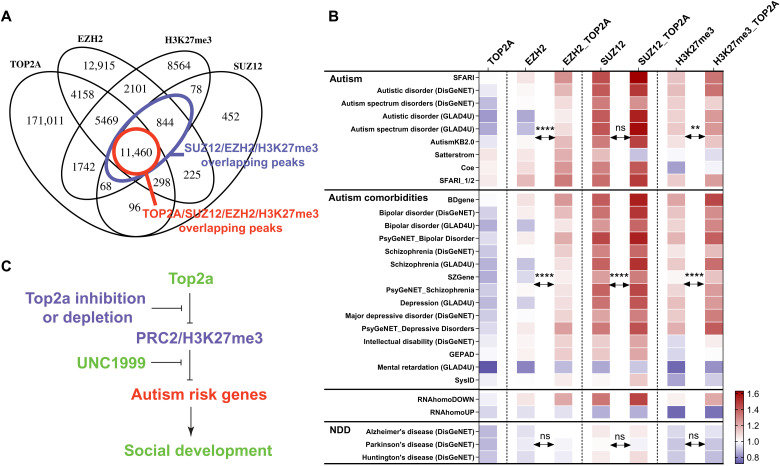
PRC2 and H3K27me3 mediate the Top2a-dependent gene regulation. (**A**) TOP2A binding peaks substantially overlap with EZH2, SUZ12, and H3K27me3. (**B**) Among all EZH2, SUZ12, and H3K27me3 binding peaks, those that overlap with TOP2A are more enriched for autism and its comorbidities risk genes and can4Dn but not for can4Up and neurodegenerative disorder risk gene sets. Significance was calculated by paired *t* test. NDD, neurodegenerative disorders. SFARI_1/2, high-confidence SFARI genes with a gene score of 1 or 2. (**C**) Working model for how PRC2 and H3K27me3 mediate the Top2a-dependent gene regulation and social development. **P < 0.01 and ****P < 0.0001.

We conducted ORA analyses to identify upstream regulators that are significantly enriched in the promoter regions of can4Dn genes but not can4Up genes. We also analyzed SFARI genes in parallel as a representative set of autism risk genes. Notably, by analyzing a curated library based on published chromatin immunoprecipitation sequencing (ChIP-seq) data [ChIP-X (*49*)], we found that the SFARI genes and can4Dn are targeted by a highly similar set of upstream regulators, especially the transcription factor SUZ12 and its associating partner EZH2 (Fig. 6A). In parallel, we analyzed a published human TOP2A ChIP-seq dataset to investigate whether TOP2A selectively binds to autism risk genes (*50*) but did not find enrichment of autism risk genes in TOP2A target genes in human K562 cells (Fig. 7B, first column). This is consistent with the traditional view of Top2a’s role as a “housekeeping” gene with no known selectivity for autism risk genes, although a Top2a ChIP-seq experiment in neurons or developing brains of zebrafish will be needed to confirm this finding. Given that Top2a binding is not enriched in autism risk genes but Top2a-dependent regulation is, we hypothesized that some of the common upstream regulators for SFARI genes and can4Dn may function as specificity factors for Top2a, enabling its influence to be heightened in autism risk genes.

To validate whether this set of upstream regulators is functionally associated with the autism risk genes found in can4Dn, we identified 70 genes that are shared by the can4Dn genes and a previously published list of validated autism risk genes, which we called the Satterstrom genes (*8*) (Satterstrom-can4Dn), and performed a protein-protein interaction (PPI) analysis to identify associations among the 15 upstream regulators shared by SFARI genes and can4Dn genes, Top2a, and the Satterstrom-can4Dn genes (fig. S10). We found that the 15 upstream regulators and Top2a are well integrated into the PPI network of Satterstrom-can4Dn genes. *k*-means clustering divided this network into two PPI clusters, and all 15 upstream regulators and Top2a are found in one of the clusters that is enriched in genes associated with chromatin modification and transcriptional regulation, whereas the other cluster is enriched in genes associated with synaptic activity regulation.

We assessed the specificity of this similarity by running a blast-style search (UES-blast) against control gene sets from a reference dataset consisting of DisGeNET, GLAD4U, and an RNA-seq gene set library, RNAseqGEO (*38*, *39*, *49*), to identify gene sets with the lowest blast scores (highest similarity rankings) to a query gene set (Fig. 6B). Using SFARI genes as a query gene set for UES-blast, we found that can4Dn, together with several independently curated autism risk gene sets, ranks among the highest, while can4Up ranks much lower. Removing overlapping SFARI genes from can4Dn (can4Dn-SFARI) does not strongly affect its ranking, indicating that the overlapping SFARI genes were not the sole contributors to their rankings. Autism comorbid conditions also rank high on the list, except for intellectual disability (Fig. 6C and fig. S11A). We also acquired several neurodegenerative disorder risk gene sets including Alzheimer’s disease, Parkinson’s disease, and Huntington’s disease from DisGeNET as negative controls because of their neurological nature, late onset, and lack of comorbidity with autism; these control gene sets rank relatively low on the list as expected. Using t-distributed stochastic neighbor embedding analysis, we found that can4Dn, SFARI, and other autism risk gene sets tightly cluster together compared to the control disease gene sets in DisGeNET (Fig. 6D) or the entire reference dataset (fig. S12). These results suggest that the Top2a-dependent can4Dn and autism risk genes share a very similar and unique set of upstream regulators.

Among the strongly associated upstream regulators, we found four that are shared by can4Dn and SFARI genes, but not by can4Up, including SUZ12, EZH2, REST, and SMAD4 (Fig. 6B). Intriguingly, SUZ12 and EZH2 have been reported to bind to TOP2A (*51*). Because both SUZ12 and EZH2 are core components of the PRC2 complex, a histone methyltransferase, we explored the enrichment of histone modifications in our gene sets using ORA and two curated libraries containing published ChIP-seq data gathered from the ENCODE and Roadmap Epigenomics projects (*49*). PRC2 methylates histone H3 on lysine-27 (i.e., H3K27me3). We found that can4Dn and SFARI genes, but not can4Up, are highly enriched in genes marked by H3K27me3 but not by the other 29 histone markers examined (Fig. 6E). We calculated a H3K27me3 score to quantify this enrichment (Materials and Methods). can4Dn and autism risk gene sets give the highest H3K27me3 scores when compared to control gene sets from the reference dataset, while can4Up scores zero as expected (Fig. 6F and fig. S11B). Autism comorbid conditions, except for intellectual disability, also give high H3K27me3 scores.

### PRC2 and H3K27me3 mediate Top2a-dependent gene regulation

We compared genome-wide binding patterns of TOP2A and PRC2 using published ChIP-seq data (*50*, *52*) in human K562 cells and found that among the triple cobinding sites of SUZ12, EZH2, and H3K27me3, >93% were also bound by TOP2A (Fig. 7A). In addition, while TOP2A binding peaks alone are not enriched for autism risk genes (Fig. 7B, first column), overlapping TOP2A binding peaks with EZH2, SUZ12, and H3K27me3 binding peaks enriched their selective targeting to autism risk genes and can4Dn but not to can4Up and the neurodegenerative disorder gene sets (Fig. 7B). These results suggest that the selective regulation of autism risk genes by Top2a may be achieved through its interplay with PRC2 and H3K27me3.

While H3K27me3 functions as a gene silencing mark, Top2a binding is strongly associated with open chromatin (*50*, *53*). A formaldehyde-assisted isolation of regulatory elements sequencing (FAIRE-Seq) analysis revealed that Top2a inhibition by ICRF-193 in mouse embryonic stem (ES) cells results in reductions in chromatin accessibility (*53*), demonstrating a role for Top2a in maintaining open chromatin. These results suggest an antagonistic relationship between Top2a and H3K27me3. We therefore reasoned that Top2a inhibition may serve to derepress PRC2 on genes contributing to social development, whereas this effect might be mitigated by a PRC2 inhibitor (Fig. 7C).

**Fig. 8.**
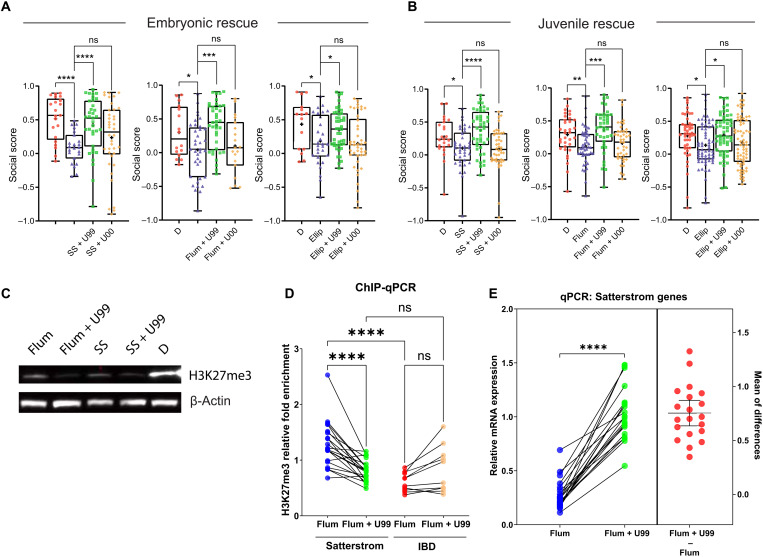
Social deficits induced by Top2a inhibition can be rescued by inhibiting PRC2. (**A**) Embryonic (0 to 3 dpf) cotreatment of 10 μM UNC1999 (U99) with 100 μM sodium salicylate (*n* = 35), 15 μM flumequine (*n* = 37), and 0.5 μM ellipticine (Ellip; *n* = 44) rescues social deficits caused by sodium salicylate (*n* = 23), flumequine (*n* = 37), and ellipticine (*n* = 29) alone, respectively, compared to DMSO control (D; left to right: *n* = 21, 16, and 15). UNC1999’s inactive analog UNC2400 (U00) does not rescue social deficits (left to right: *n* = 38, 19, and 42). (**B**) Social deficits caused by embryonic treatment of sodium salicylate (*n* = 38), flumequine (*n* = 45), and ellipticine (*n* = 61) compared to DMSO (left to right: *n* = 23, 34, and 47) are rescued by overnight exposure to UNC1999 (left to right: *n* = 44, 39, and 56) but not UNC2400 (left to right: *n* = 42, 41, and 67) at the juvenile stage (20 to 21 dpf). (**C**) Western blot shows the global down-regulation of H3K27me3 in 3 dpf larvae by flumequine and sodium salicylate compared to DMSO control. Cotreatment of U99 further reduced H3K27me3, as expected. (**D**) ChIP-qPCR shows significant differences in the relative fold enrichment of H3K27me3 in Satterstrom versus IBD genes in 3 dpf larvae treated with flumequine. The relative H3K27me3 level is higher in Satterstrom (blue) than in IBD (red) genes. H3K27me3 was reduced in Satterstrom (green) and increased in IBD (brown) genes by U99. Each dot represents data for a single gene. (**E**) qPCR shows the down-regulation of Satterstrom genes in the brain of adult zebrafish that received embryonic treatment of flumequine (using the same data shown in Fig. 5E) compared to DMSO control. Embryonic cotreatment of U99 rescued gene expression. Differences in mRNA expression between these two samples (flumequine + U99 − flumequine) are shown as red dots; horizontal bars represent the mean and SD of the differences. Significance is calculated using paired *t* test. *P < 0.05, **P < 0.01, ***P < 0.001, and ****P < 0.0001.

To test this hypothesis, we added the Ezh2 inhibitor UNC1999 and its inactive control compound UNC2400 (*54*) alongside Top2 inhibitors during embryonic exposure and found that UNC1999 effectively ameliorates social deficits caused by flumequine (a fluoroquinolone antibiotic), ellipticine (a Top2 poison), and sodium salicylate (a Top2a-selective Top2 catalytic inhibitor), while UNC2400 shows no effective rescue (Fig. 8A). Perhaps more strikingly, we found that overnight exposure to UNC1999, but not UNC2400, the night before Fishbook test effectively rescued social deficits in juvenile fish treated with Top2 inhibitors during embryonic development (Fig. 8B). These findings suggest that a substantial portion of the social deficit phenotype may be attributed to elevated H3K27me3 that was introduced by Top2a inhibition at the embryonic stage and persisted to the juvenile stage.

Western blot showed a global down-regulation instead of up-regulation of H3K27me3 upon Top2 inhibitor treatment (Fig. 8C and fig. S13), which is consistent with a previous observation in mouse ES cells upon Top2a knockdown (*51*). We propose that, instead of functioning through global regulation of H3K27me3, Top2a may function by modulating the relative level of H3K27me3 between autism risk genes and non-autism risk genes. To examine this hypothesis, we conducted a ChIP-qPCR experiment to compare the relative fold enrichment of H3K27me3 in the promoter regions of zebrafish orthologs of the 13 highest-ranking Satterstrom genes (*8*) (table S2) versus 8 high-confidence inflammatory bowel disease (IBD) risk genes as controls (table S3) (*55*) upon Top2a inhibition. We found that 3 dpf embryos treated with flumequine or sodium salicylate have elevated H3K27me3 in the selected Satterstrom genes compared to the IBD genes (Fig. 8D and fig. S14A). Cotreating the embryos with UNC1999 down-regulated H3K27me3 in the Satterstrom genes, while it up-regulated H3K27me3 in the IBD genes, therefore restoring a balanced level of H3K27me3 between the Satterstrom and IBD genes (Fig. 8D and fig. S14A). Consistent with their up-regulated H3K27me3, the Satterstrom genes are persistently down-regulated in the brain of adult zebrafish that received embryonic treatment of flumequine or sodium salicylate, and this down-regulation in gene expression is rescued by embryonic cotreatment of UNC1999 (Fig. 8E and fig. S14B).

## DISCUSSION

Association studies have now identified hundreds of genetic variants that each contribute a small amount of autism risk (*7*–*9*, *56*). Less than 1% of autism cases are caused by a single-gene defect (*57*). Given the large number of genes involved and the inability of most of these genes to individually explain more than a small amount of increased risk, one might ask whether the many autism risk genes are regulated independently or are coordinately regulated in some way. Evidence of coordination might point toward shared mechanisms of autism etiology and gives hope that autism spectrum disorders of diverse genetic backgrounds might be treatable by targeting their shared mechanisms.

Although genetic factors are clearly important contributors to ~60% of autism risk, environmental factors appear to explain an additional ~40% of risk (*10*, *11*). Despite the importance of identifying environmental factors contributing to autism, few have been identified. Interest in identifying environmental risk factors of autism is intense; even a fraudulent claim linking vaccinations and autism prompted lasting changes in vaccine utilization (*58*). The difficulty of linking environmental factors with neurobehavioral development may be largely attributed to the massive number of environmental factors and the limited throughput of current research methodologies. Only a fraction of the chemicals in our environment have been examined for potential adverse effects on neurobehavioral development (*59*). Current experimental methods mainly rely on rodent models. Although these models can often recapitulate complex behavioral phenotypes that relate to human diseases, the time, space, and financial resources required can be prohibitive for conducting large-scale experimental screens. Models enabling large-scale testing of the impact of small molecules on social development are needed.

The zebrafish has an evolutionarily conserved social brain network (*12*). Many components of this network have been studied in rodent models for social-related behaviors, such as social reward (*60*), aggression (*61*), mating (*62*), social learning (*63*), and social memory (*64*), which paved the way for using zebrafish to investigate social behavior and autism. The Fishbook platform enables systematic evaluation of environmental chemicals to identify those affecting social behavior development. In a systematic small-molecule screen, we identified Top2a inhibitors as profound disruptors of social development in zebrafish and mice. Disruption of Top2a preferentially alters the expression of many of the genes previously associated with autism in humans. Hence, Top2a may serve as a common regulatory factor controlling a large set of genes known to confer autism risk and, hence, may also serve as a link between genetic and environmental contributors to autism.

Prior studies have implicated Top2a in autism via different approaches. One study identified *TOP2A* as a member of a core autism risk gene network consisting of 234 genes by analyzing whole-exome sequencing data (*24*). Another revealed *TOP2A* as 1 of the 23 hub genes related to brain maldevelopment in autism toddlers (*25*). The Top2 inhibitor ICRF-193 has been shown to inhibit the transcription of extremely long genes that are associated with autism in cultured mouse cortical neurons, ES cells, and ES cell–derived neurons (*23*). In corroboration of both this finding and ours, a FAIRE-Seq analysis found that ICRF-193 caused a reduction in chromatin accessibility in ES cells, especially in long silent genes (*53*). Several Top2 inhibitors were found to reactivate the dormant allele of the autism and Angelman syndrome gene *Ube3a* in primary cortical neurons from mice by reducing the transcription of an imprinted antisense RNA, *Ube3a-ATS* (*22*).

Fluoroquinolones are generally avoided during pregnancy because of their association with developmental toxicity in animals. Despite this consensus, fluoroquinolones are occasionally prescribed to pregnant women (*65*), likely because of providers being unaware of the pregnancy or being unaware of the potential risk. The results presented here may call for additional investigation into whether prenatal fluoroquinolone exposure in humans increases autism risk. It is noteworthy that a number of known or suspected environmental risk factors of autism are Top2 inhibitors, such as bisphenol A (*15*), polychlorinated biphenyls (*16*, *66*), chlorpyrifos and chlorpyrifos oxon (*17*), and genistein (*67*). Top2 inhibitors are widely present in the environment, and their possible link to autism has previously been proposed (*68*). Chemicals with Top2-inhibiting activities that are present in the environment but have not yet been clearly associated with autism include flavonoids (*69*), alternariol (*70*), *Ginkgo biloba* leaf extract (*71*), and lignans (*72*), to name a few. Environmental pollution by drugs with Top2 inhibitor activities has also been reported (*73*, *74*). Nevertheless, it is not known whether Top2a inhibition is a meaningful contributor to autism prevalence in humans. Further epidemiological studies will be needed.

It is perhaps unexpected that Top2a, an enzyme often regarded as performing housekeeping functions, might have a specific effect on the development of sociality. As described here, Top2a binds DNA throughout the genome, with no detected preference for genes associated with autism. The situation changes, however, when considering Top2a colocalized with the PRC2. Colocalization of Top2a, PRC2, and the PRC2-deposited epigenetic mark H3K27me3 is highly enriched among autism risk genes, including those found through human GWASs and those identified in zebrafish in this study. Hence, Top2a appears to influence the expression of diverse genes that together regulate social development by antagonizing PRC2/H3K27me3-mediated gene silencing. In alignment with this hypothesis, mutations in both H3K27 demethylases, KDM6A and KDM6B, have been associated with autism (*75*, *76*), linking defects in a H3K27me3-antagonizing mechanism with autism etiology. Why so many autism risk genes would be co-regulated by the Top2a/PRC2 mechanism is unclear. One possibility is that many genes are required for successful social development and that being able to simultaneously regulate their expression during development is advantageous.

The fact that the two structurally similar and functionally overlapping isoforms of Top2, Top2a and Top2b, play distinct roles in regulating the development of sociality suggests that differences in their expression patterns might offer an explanation to their functional divergence. While the expression of Top2b is restricted to nondividing postmitotic cells, Top2a is primarily expressed in progenitor cells (*53*): Its expression increases two- to threefold during G_2_-M and is orders of magnitudes higher in rapidly proliferating than in quiescent cells. This suggests that understanding the acute or prolonged impacts of Top2a inhibition on progenitor cells might be the key to unravel the mystery of Top2a’s mechanism of action.

The effects of Top2a inhibition on sociality appear to be durable: Zebrafish and mice treated transiently with Top2a inhibitors during development exhibit social deficits weeks later in the case of zebrafish and months later in the case of mice. This durability may reflect a permanent developmental defect, for example, if Top2a inhibition during development alters the structure of the brain, for example, by inhibiting progenitor cell proliferation, in such a way that normal sociality cannot be recovered even after Top2a activity is restored. Alternatively, the social deficits may reflect gene expression changes, such as in genes that regulate neurotransmission or synaptic function, that are preserved by epigenetic marks but might theoretically be reversed by a second epigenetic intervention. For example, epigenetic changes in progenitor cells caused by Top2a inhibition might be inherited by their cellular offspring and result in lasting changes in gene expression, which negatively affect neuronal functions that relate to social behavior. For future investigations, the two hypotheses explaining the durable inhibition of sociality may be examined together using a single-cell multiomic approach: The potential developmental defects caused by progenitor inhibition could be revealed by brain cell population analysis through single-cell RNA-seq, whereas the potential epigenetic changes inherited by cellular offspring from progenitor cells and their effects on gene expression could be assessed by a combination of single-cell ChIP-seq and single-cell RNA-seq experiments. We found that manipulation of the Top2a/PRC2 pathway using the Ezh2 inhibitor UNC1999 could rescue social deficits caused by Top2 inhibition, not only when cotreated during development but also even in older animals. In particular, overnight exposure to UNC1999 rescued social deficits caused by embryonic Top2 inhibition, suggesting that a short-term modulation of this pathway at the juvenile stage may be sufficient to boost prosocial gene expression. This phenomenon, if conserved in mammals, may offer hope that Top2a-mediated social deficits might be reversible through epigenetic reprogramming.

While the total level of H3K27me3 was reduced by Top2 inhibitors (Fig. 8C and fig. S13), we found that H3K27me3 was selectively elevated in Satterstrom genes, a representative set of high-confidence autism risk genes (Fig. 8D and fig. S14A). How Top2 inhibition produces this selective elevation of H3K27me3 requires further investigation, but it does suggest that Top2a may influence methylation in two different ways. For example, Top2a may play an indirect role in supporting methylation activity generally through its function in maintaining genomic structure and accessibility during DNA replication and transcription. Top2a binding is strongly associated with open chromatin (*50*, *53*) and required for maintaining chromatin accessibility (*53*). Upon Top2a inhibition, as chromatin structure and accessibility are altered, the overall H3K27 trimethylation may be affected genome wide, resulting in the observed global reduction of H3K27me3. At the level of individual genes, however, including many of the genes from the Satterstrom set, it appears that Top2a performs a different function, antagonizing PRC2 activity. We observed that PRC2 selectively binds to autism risk genes (Fig. 7B), and the colocalization of PRC2 and Top2a is particularly enriched at those genes. Therefore, we hypothesize that inhibition of Top2 relieves repression of PRC2 at autism risk genes, promoting relative elevation of H3K27me3 in those genes compared to the rest of the genome. Further investigations are needed to examine these hypotheses.

In summary, this study reveals a crucial role for Top2a in promoting the development of social behavior in zebrafish and mice. Our results suggest that Top2a is indispensable for maintaining an upstream regulatory network that selectively controls the expression and epigenetic status of a large subset of autism risk genes. Top2a likely functions by antagonizing PRC2 and H3K27me3-mediated gene silencing. Further analyses are warranted to examine potential links between prenatal exposures to Top2-inhibiting chemicals and autism risk in humans through epidemiological and toxicological approaches.

## MATERIALS AND METHODS

### The Fishbook system and social score

The basic unit (test arena) of this system is a 3D printed, 10-mm-deep, 8.5-mm-wide, and 80-mm-long rectangular chamber. Each chamber is divided into three compartments by two transparent acrylic windows (1.5 mm thick): a 60-mm-long middle testing chamber to place the test subject and two 8.5-mm-long end chambers to place the social stimulus fish or remain empty, respectively. The walls are 1.5 mm thick. The 3D printing model was created using Tinkercad (Autodesk). These arenas were 3D printed using white polylactic acid at 100% infill. Printed arenas were glued onto 3/16″ thick white translucent (43% light transmission) acrylic sheets (U.S. Plastic) using a silicone sealer (Marineland). Transparent acrylic windows were precision-cut to 8.5 mm by 10 mm pieces using a laser cutter and inserted into printed slots in the arena and fastened using a silicone sealer.

The key component of our imaging system is a 322-mm-diameter bi-telecentric lens (Opto Engineering) with an infrared (850-nm) band-pass filter (Opto Engineering). Videos were taken by a Blackfly 5.0 MP Mono USB3 Vision camera (Point Grey) at 7.5 frames/s. Ambient light was provided using white light-emitting diodes (LEDs; Environmental Lights). The arenas were illuminated from below with infrared (850-nm) LEDs (Environmental Lights). Infrared LEDs were cooled by a heat sink (HS Marston). Structural supports and enclosure were custom built using parts purchased from Thorlabs, McMaster Carr, and U.S. Plastic.

Test subjects were individually placed inside each test chamber using a plastic transfer pipette with its tip cut off to widen the opening. Their visual access to social stimulus fish was temporarily blocked by a 3D printed white comb-like structure placed in front of the social stimulus compartment (fig. S1, E and F). Once all test subjects were placed into the test arena array, the array was placed inside the imaging station, and the combs were removed to visually expose the social stimulus fish to the test subjects. After a 5-min acclimation period, a 7.5-min (for screening and validation) or 10-min test session was video recorded.

Videos were streamed through the software Bonsai (*77*). Videos were analyzed in real time during recording, and the frame-by-frame *x* and *y* coordinates of each fish relative to its own test compartment were exported as a CSV file. Data were analyzed using custom scripts (Python) to calculate social scores and generate tracking plots. Social score was defined as a fish’s average *y*-axis position for all frames. We designated the middle of each test chamber as the origin of the *y* axis. We then assigned a value of 1 to the end of the chamber close to the social stimulus fish and a value of −1 to the other end of the chamber, which is close to the empty control compartment. Therefore, in this coordinate system, all social scores have values between −1 and 1. A higher social score demonstrates a shorter average distance between a test fish and a social stimulus fish during a test, which suggests a stronger social preference.

### Zebrafish embryonic chemical treatment

For high-throughput screening, fertilized eggs were transferred into 96-well, square-well plates with 12 eggs per well. Each well was filled with a 500-μl-volume E3 medium. E3 medium was supplemented with penicillin/streptomycin to prevent bacterial contamination due to accidental or drug-induced embryo deaths in a crowded space. No penicillin/streptomycin was used for subsequent validation experiments. Compounds in the Prestwick chemical library [all dissolved in dimethyl sulfoxide (DMSO)] were screened at 1:500 dilution for a final concentration of 20 μM. Negative controls were treated with an equal volume of DMSO. Each compound was added to two wells. All or 50% of medium and drugs were changed at 1 and 2 dpf, respectively. Larvae typically hatch between 2 and 3 dpf. At 3 dpf, all hatched and live but unhatched larvae treated by the same compound were collected and transferred to a petri dish containing fresh E3 medium without penicillin/streptomycin. At 5 to 7 dpf, larvae from each petri dish were transferred to a separate nursery tank to be raised to 3 weeks of age for Fishbook assay.

For individual compound treatments, each chemical’s general toxicity level was first estimated by measuring the overall embryonic lethality after 3 days of treatment (0 to 3 dpf). Most biologically active small molecules are toxic to zebrafish embryos when applied at high doses, and toxicity is often a good benchmark assessment of effective compound exposure. Each compound was first applied to the embryos from 0 to 3 dpf by dissolving in E3 medium at a wide range of doses, often ranging from 0.1 μM to 1 mM with 10-fold serial dilutions. Two to three additional rounds of assessments were then conducted to narrow down the dose ranges and identify the median lethal dose (LD_50_) of each compound. Once toxicity is assessed, compound treatments began at LD_50_ and simultaneously increased and decreased two to three times serially by twofold (at LD_50_ × 4, LD_50_ × 2, LD_50_/2, LD_50_/4, etc.). We have also found that some compounds, when applied at a certain dose, showed little to no toxicity at the embryonic stage but caused massive lethality at a later stage once these drug-treated larvae entered nursery. For these compounds, further adjustments of compound doses were required to identify their new LD_50_ values with nursery survival taken into consideration. Most inhibitors tested effectively induced social deficits when applied at LD_50_. If a compound showed no effect in inducing social deficits at lower doses, at least one dose at >LD_50_ must be successfully tested before a conclusion could be made regarding the compound’s effectiveness. All compounds were dissolved in DMSO (Sigma-Aldrich) or water. The final DMSO concentration was never higher than 1%.

The Peterson laboratory previously found that the logarithm value of a compound’s octanol:water partition coefficient (logP) strongly correlates with the compound’s embryonic permeability (*78*). A logP value higher than 1 predicts good absorption, whereas compounds with a logP value lower than 1 are often poorly absorbed by the zebrafish embryo. This principle has been confirmed by the author’s own observations, as, in our hands, a compound with logP values lower than 1 often failed to cause any detectable toxicity even when applied at extremely high doses in E3 medium. This problem can be overcome by injecting compounds directly into embryos at the one- or two-cell stage (*78*). Therefore, compounds with logP values lower than 1, including ICRF-193, etoposide, bleomycin, and hydroxyurea, were directly injected into embryos. Their respective dosages were determined by first injecting a range of doses, often ranging from 0.1 μM to 10 mM with 10-fold dilutions in between, to assess their general toxicity levels as quantified by the percentage of embryonic lethality induced at each dosage. The doses were then narrowed down and refined to identify the LD_50_ value of each compound. Last, each drug was injected at its LD_50_ dose and at several doses higher and lower (with twofold differences) than the LD_50_ value.

### High-throughput screening and validation

A total of two rounds of screening and one round of validation were performed. The first round exposed 24 embryos to each compound. Treatment groups with social scores lower than 0.1 were identified. These compounds were then hand-picked for the second round of screening, which exposed 48 embryos to each compound. Treatment groups that again inhibited social score to <0.1 were identified. These compounds were purchased from commercial sources and used to treat 100 embryos. Compounds that significantly reduced social score compared to the DMSO control were identified as hits.

Three-week-old fish grown in our nursery are typically ~1 cm long and very similar in size. We generally do not select for size before Fishbook testing to avoid selection bias. However, we do occasionally remove a very small percentage of fish (<1%) that are exceedingly small (<5 mm) and appear sickly before testing.

### Chemical library and other compounds

The Prestwick library (Prestwick Chemical) contains 1120 approved drugs dissolved in DMSO at a stock concentration of 10 mM. Other compounds were purchased from the following sources: from Cayman Chemical: flumequine, oxolinic acid, etoposide, mitoxantrone (hydrochloride), ellipticine, bleomycin (sulfate), hydroxyurea, actinomycin D, triptolide, and UNC1999; from Sigma-Aldrich: lomefloxacin hydrochloride, ofloxacin, and sodium salicylate; from ApexBio: amsacrine; from Santa Cruz Biotechnology: ICRF-193, sobuzoxane, and aclarubicin (aclacinomycin A); from Tocris: UNC2400. All individually purchased compounds were dissolved in DMSO or water. Chemical structures were generated using PubChem Sketcher.

### Zebrafish juvenile chemical treatment and Fishbook testing

For the juvenile rescue experiments, 20 dpf zebrafish were collected from nursery tanks. Fish that received the same treatment at the embryonic stage but raised in different tanks were pooled together and then sorted into deep petri dishes (such as a 25-mm-deep petri dish) containing 40 ml of E3 medium mixed with rotifers as fish feed and 5 μl of AmQuel (Kordon) to remove harmful ammonia excreted by fish. Fish (10 to 15) were sorted into each dish. Compounds were then added to each dish. Dishes were incubated at 28°C in a larvae incubator overnight. At 21 dpf, each dish is fed lightly with rotifers in the morning. Fishbook assay was conducted ~1 to 2 hours after feeding. Immediately before plating fish in each petri dish into the Fishbook test arena, the content of the petri dish is poured through a nylon tea strainer so that all liquid passes through and fish are kept in the tea strainer. The tea strainer containing the fish is then consecutively dipped into three petri dishes containing E3 to wash the residual chemical away from the fish. The fish is then poured into a petri dish containing clean E3 and transferred into the Fishbook test arena using a plastic transfer pipette for testing.

### Site-directed mutagenesis

Constructs containing full-length human *TOP2A* complementary DNA sequence were purchased from Genecopoeia. Site-directed mutagenesis was conducted using the QuikChange Site-Directed Mutagenesis Kit (Agilent). The forward and reverse mutagenic primers for *TOP2AY165S* are 5′-aatttggctccagagccatttcgaccacctgtcact-3′ and 5′-agtgacaggtggtcgaaatggctctggagccaaatt-3′.

### Morpholino injection and validation

Splice-blocking morpholinos targeting zebrafish Top2a (5′-GACATTCATCATAAACTCACCCAGA-3′) and Top2b (5′-ATGCAGTAATCTTACCAAGGATCTC-3′) were purchased from Gene Tools. To validate their knockdown efficiencies, injected embryos were collected for mRNA extraction using the RNeasy Mini Kit (QIAGEN) at 1 dpf, followed by reverse transcription using the QuantiTect Reverse Transcription Kit (QIAGEN) and PCR using primers that flank the targeted exons. Top2a-MO knockdown efficiency was examined using the forward and reverse primers 5′-GGCTCTCAGTAAGCCCAAGAA-3′ and 5′-TACTGGAGGTCAGGAGTTGGC-3′, with the full-length amplicon predicted to be 401 base pairs (bp) and splice-blocked amplicon to be 310 bp long. Top2b-MO knockdown efficiency was examined using the forward and reverse primers 5′-AAATGAATGGCCGGGGAGATG-3′ and 5′-TGATGGTTGTCATGTTCTTGTCTCT-3′, with the full-length amplicon predicted to be 297 bp and splice-blocked amplicon to be 206 bp long. The toxicity of the morpholinos was examined, and the doses were determined by following a protocol described in the “Zebrafish embryonic chemical treatment” section.

### mRNA overexpression and validation

mRNA in vitro syntheses were conducted using the mMESSAGE mMACHINE T7 Transcription Kit (Invitrogen). Linearized constructs containing *TOP2A* and *TOP2AY165S* genes were used as templates. Following synthesis, mRNAs were A-tailed using *Escherichia coli* Poly(A) Polymerase (NEB) to improve translation efficiency. *TOP2A* and *TOP2AY165S* mRNAs were injected to the yolk of one- to two-cell stage embryos. For validation of protein overexpression, ~200 to 300 embryos were dechorionated 3 to 6 hours after injection by 5 min of incubation in pronase (1 mg/ml; Roche) in E3 medium in a 2% agarose-coated petri dish and washed with excess amount of fresh E3. Dechorionated embryos were transferred to a 1.5-ml tube and deyolked by pipetting up and down three to five times using a P200 tip in 200 μl of ice-cold phosphate-buffered saline (PBS). Ice-cold PBS (1 ml) was added, and the resulting mixture was centrifuged at 300*g* for 30 s. The supernatant was discarded, and the cell pellet was washed with an additional milliliter of ice-cold PBS.

### Western blot

Cells (0 dpf mRNA overexpression samples) or larvae (3 dpf Top2 inhibitor–treated larvae) were lysed in radioimmunoprecipitation assay buffer (Santa Cruz Biotechnology) supplemented with a cOmplete Mini EDTA-free protease inhibitor tablet (Roche), 5 mM sodium orthovanadate (New England Biolabs), and 10 mM sodium fluoride (Sigma-Aldrich) for 50 min on ice with vortexing every 10 min. The resulting lysate was centrifuged at 14,000*g* for 15 min at 4°C. The entire volume of the supernatant was saved and subsequently combined with 5× Laemmli buffer before gel loading for Western blot analysis. Protein concentration was measured by Bradford (Bio-Rad) assay. Anti-TOP2A (Cell Signaling Technology, D10G9), anti-H3K27me3 (Cell Signaling Technology, C36B11), and anti–β-actin (Cell Signaling Technology, D6A8) antibodies were used for detection. Signal was detected by enhanced chemiluminescence.

### Zebrafish mutagenesis and genotyping

Frameshift mutations were engineered into the *nrg1* and *disc1* genes of TuAB strain zebrafish at exons 2 and 4, respectively, using CRISPR-Cas9–mediated genome editing. The CRIPSR guide RNA spacer sequences were 5′-GGCCGAGGGAGTGGTGCTGG-3′ (*nrg1*) and 5′-GGATACATGCGGTCTGAGCC-3′ (*disc1*). The primers used for genotyping were 5′-TAGGCCGAGGGAGTGGTGCTGG-3′ and 5′-AAACCCAGCACCACTCCCTCGG-3′ for *nrg1* and 5′-TAGGCTCAGACCGCATGTATCC-3′ and 5′-AAACGGATACATGCGGTCTGAG-3′ for *disc1*. A 20-bp deletion (GGATACATGCGGTCTGAGCC) was detected in exon 2 of *disc1* gene, resulting in a premature stop codon (fig. S1E). A 22-bp deletion (CCAGCACCACTCCCTCGGCCAA) was detected in exon 4 of *nrg1* gene, also resulting in a premature stop codon (fig. S1F).

The mutant line *can4* was genotyped using the forward and reverse primers 5′-CTGCAGAAACCCTGTTAAG-3′ and 5′-AGGGGATTGACCTCTCGTTG-3′ (*32*). The mutant line *noto* was genotyped using the forward and reverse primers 5′-TCGGTTCCAAATGTGCTCTCT-3′ and 5′-AGCCTTGCAAGCCCTAATCAT-3′ (*33*). PCR and subsequent Sanger sequencing were performed using the same primers. Genomic DNA extracted from whole fish following Fishbook assays was purified (Zymo Research) before PCR to remove PCR inhibitors in the fish tissues.

### Optomotor response assay

Optomotor response assay was conducted by following an established protocol (*29*). Briefly, five 21 dpf fish were placed in a 10-cm petri dish with a cylinder (two 50-ml tube caps taped together) placed in the middle. The petri dish was placed on a computer screen. After a 1-min acclimation period, a clockwise rotating pattern was displayed for 30 s, followed by a 30-s break. Another counterclockwise rotating pattern was then displayed for 30 s, followed by another 30-s break. A blinded observer counted the number of larvae swimming toward the same direction of the stimulus at the 20- and 30-s marks during each period (the directions of stimulus during the break periods are set to be similar to the direction during the stimulus period before the break). An average of the two counts for each period was treated as a data point.

### Mouse prenatal injection

Assessing pregnancy according to the presence of vaginal plugs can be unreliable (*79*). We therefore assessed pregnancy on the basis of weight gain following a reported protocol (*79*) with modifications. We considered the day after overnight pairwise breeding to be potentially 0 dpc. Females were first weighed at 0 dpc, then single-housed until 7 dpc when they were weighed again. Females with weight gains >1.0 g were considered pregnant. Drug and vehicle control were injected daily through intraperitoneal injection from 7 to 18 dpc. Births were given on 19 or 20 dpc. ICRF-193 was first dissolved in 100% ethanol (Thermo Fisher Scientific) and then diluted in saline (Baxter) to a final concentration of 0.01 μg/μl. Diluted drug was injected at a volume of 10 μl/g body weight to achieve a final dosage of 0.1 mg/kg.

### Social interaction assay

Social interaction was examined as described previously (*35*) using 2-month-old mice. Briefly, each adult mouse was introduced into a neutral and unfamiliar cage. After a 5-min acclimation period, a foreign age-, sex-, and weight-matched untreated mouse (from separate litters) was introduced into the cage as a social stimulus. Both experimental and untreated females were assayed at the metestrus or diestrus stages to minimize the influence of estrous cycle on social behavior. The test mouse was marked by an odorless black marker at the tail to differentiate from the social stimulus mouse. Testing sessions lasted for 5 min and were manually scored for social interactions later. Durations of each social interaction were timed using the software BORIS (*80*). All mice were scored blind to their treatment conditions. Social interaction was defined as any period of time in which the test mouse was actively investigating the social stimulus mouse. Investigations included sniffing of the social stimulus mouse toward its facial, abdominal, and anogenital areas; grooming; or closely pursuing the stranger as it explored the cage; investigation of the test mouse by the social stimulus mouse was not scored.

### Novel object investigation assay

The novel object test was performed in a neutral and unfamiliar cage using 8-month-old mice in the exact same manner as the social interaction assay, except with a wooden block introduced to the cage after the acclimation period instead of a social stimulus mouse. Total time spent investigating the object was quantified over a 5-min period. Objects were covered with tape during the test; tape was thoroughly removed and replaced in between tests to remove odor traces.

### USV assay

Vocalizations were tested in pups on postnatal day 7 or 8. Cages were acclimatized to the room for 30 min. Pups were individually placed on the test platform in a soundproof cabinet. USVs were measured for 5 min using a USV detector (Avisoft Bioacoustics).

### T-maze assay

T-maze assay was performed using 3-month-old mice. Each session consisted of eight consecutive trials. In each trial, the mouse was placed in the start compartment of a T-maze. After 1 min before the first trial and 15 s for the subsequent trials, the door was lifted, and the mouse was left free to explore the two arms of the maze. As soon as the animal entered (with all four paws) one of the two alternative arms (left or right), the door of that compartment was closed for 30 s to confine the animal. The animal was removed between trials, and the T-maze was quickly cleaned with 70% alcohol and dried to remove any odor traces that may affect the performance in the next trial.

### Hole-board assay

Hole-board assay was conducted in a 16-hole square apparatus as previously described (*35*) using 3-month-old mice. Each session lasted for 10 min, and the total number of head pokes was recorded. The occurrence of perseverative behaviors (manifested as the tendency to explore the same holes) was measured via the coefficient of exploratory variation, calculated as the ratio of the SD of the number of head pokes for each hole over the mean for each mouse. This index provides a quantitative estimation of the dispersion of the probability distribution of exploratory activity with regard to perseverative behaviors.

### Open-field assay

Testing was conducted using 10-month-old mice. The open field consisted of a gray plexiglass square arena (40 cm by 40 cm) enclosed by four black walls (40 cm high). On the floor, two zones of equal area were defined: a central square zone of 28.28 cm per side and a concentric peripheral zone including the area within 11.72 cm from the walls. Mice were placed in the central zone at the beginning of each assay, and their behaviors were each monitored for 10 min. Animal motions were tracked using Bonsai. Percentage of time spent in the center zone and average velocity were measured using a custom-written Python script.

### Animal husbandry

All animal husbandry and experiment protocols were approved by and carried out in accordance with the Institutional Animal Care and Use Committee at Massachusetts General Hospital or University of Utah. Fertilized eggs (up to 10,000 embryos per day) were collected from group mating of EkkWill strain zebrafish (*D. rerio*) (EkkWill Waterlife Resources). Embryos were raised in Hepes (10 mM)–buffered E3 medium at 28°C, with or without compound treatment, during the first 3 days. At 3 dpf, chorion debris was removed, and larvae were transferred into petri dishes containing fresh E3 medium. At 5 to 7 dpf, larvae were transferred into nursery tanks and raised at 28°C on a 14-hour on/10-hour off light cycle.

Male C57BL/6J mice (*Mus musculus*) were obtained from the Jackson Laboratories. Mice were group-housed at no more than five per cage and were maintained on a 12-hour light/12-hour dark cycle. The room temperature (RT) was maintained at 20° to 23°C with relative humidity at approximately 50%. Food and water were available ad libitum for the duration of the study, except during testing. All tests were conducted during the light phase of the light-dark cycle.

### Statistical analysis

Graphs were generated using GraphPad Prism or Python. Data were analyzed using two-tailed Student’s *t* test. Bonferroni correction was used to adjust for multiple comparison, except for ORA conducted using the enrichR R package. For ChIP-qPCR, significance *P* values were calculated using ordinary one-way analysis of variance (ANOVA) and Tukey’s multiple comparisons test. *P* values less than 0.05 were considered significant.

### RNA-seq and data analysis

We conducted RNA-seq on 3 dpf *can4^−/−^* mutants and their WT siblings. Sequencings and initial data processing were conducted at Huntsman Cancer Institute High-Throughput Genomics and Bioinformatic Analysis Shared Resource. We first incrossed heterozygous *can4* mutants to produce a mixture of *can4^+/−^* (heterozygous), *can4^−/−^* (homozygous), and *can4^+/+^* (WT) embryos. We then lysed 3 dpf larvae individually by rigorously pipetting in 55 μl of DNA/RNA Shield (Zymo Research) at RT followed by overnight incubation at 4°C. We extracted genomic DNA from 5 μl of lysate of each larvae using a Zymo 96 genomic DNA purification kit and genotyped each sample by PCR and Sanger sequencing. The remaining lysates were stored at −80°C while awaiting genotyping results. Once genotyped, larvae with the same genetic background were pooled for mRNA extraction using the Direct-zol RNA Miniprep Kit with TRI Reagent from Zymo Research. We prepared five biological replicates for each genetic background—*can4^−/−^* and WT—with each sample containing mRNA extracted from nine larvae. Libraries were prepared using the Illumina TruSeq Stranded Total RNA Library Prep Ribo-Zero Gold Kit. Sequencing was conducted in NovaSeq with 2 × 150 bp runs at 50 million reads per sample. Sequencing reads were aligned using STAR (*81*). Quality control was performed using MultiQC (https://multiqc.info/). Initial data processing and analyses were performed using the R packages DESeq2 (*82*) and hciR (https://github.com/HuntsmanCancerInstitute/hciR). Data were normalized using the regularized log (rlog) counts method in DESeq2. The *can4^−/−^* mutant data were compared to WT data to identify genes with significantly altered expressions. The raw and processed RNA-seq data have been deposited in the Gene Expression Omnibus (GEO) under accession number GSE181730.

### Gene sets and gene set libraries

The SFARI (*40*, *41*) gene set was acquired from the archived online SFARI gene database (https://gene-archive.sfari.org/tools/). The Satterstrom gene set was acquired from Satterstrom *et al.* (*8*). The Coe gene set was acquired from Coe *et al.* (*9*). The AutismKB 2.0 (*42*) gene set was downloaded from the AutismKB website (http://49.4.21.8:8008/). The BDgene (*43*) gene set was downloaded from the BDgene website, which is no longer accessible; we have listed all genes in the BDgene gene set in table S4. The SZGene (*45*) gene set was acquired from www.szgene.org/. The SysID (*46*) gene set was downloaded from SysID database website (https://sysid.cmbi.umcn.nl/table/human-gene-info). PsyGeNET (*44*) gene sets for depressive disorders (PsyGeNET Depressive Disorder), bipolar disorder (PsyGeNET Bipolar Disorder), and schizophrenia spectrum and other psychotic disorders (PsyGeNET Schizophrenia) were downloaded from the PsyGeNET website (www.psygenet.org/web/PsyGeNET/menu/downloads). The GEPAD (*47*) gene set was acquired from the cited publication.

The gene set libraries DisGeNET, RNAseqGEO (RNA-Seq_Disease_Gene_and_Drug_Signatures_from_GEO), ChIP-X (ENCODE_and_ChEA_Consensus_TFs_from_ChIP-X), ENCODE_HM (ENCODE_Histone_Modifications_2015), and Epigenomics_Roadmap_HM (Epigenomics_Roadmap_HM_ChIP-seq) were downloaded from the Enrichr (*49*) website (https://amp.pharm.mssm.edu/Enrichr/#stats). The GLAD4U disease gene set library was downloaded from the WebGestalt (*83*) website (www.webgestalt.org/).

### Overrepresentation analysis

ORA using DisGeNET, KEGG (KEGG_2019_Human), REACTOME (Reactome_2016), and GO term (biological processes, molecular function, and cellular component) libraries (GO_Biological_Process_2018, GO_Molecular_Function_2018, and GO_Cellular_Component_2018) was performed in R using the enrichR package (https://cran.r-project.org/web/packages/enrichR/index.html). Results were ranked on the basis of the adjusted *P* value.

Other ORA assays were performed in R using a one-sided Fisher’s exact test with 95% confidence calculated according to the R function fisher.test, and Bonferroni correction was used to adjust for multiple comparison in these tests. The total number of human coding genes was set at 20,438 at the time of analysis (GRCh38.p13; https://uswest.ensembl.org/Homo_sapiens/Info/Annotation).

Permutation analysis was conducted by randomly selecting 5000 genes from all 14,989 human orthologs of zebrafish genes for each round of permutation. The null distribution consisted of odds ratios calculated for 1000 permutations. The 14,989 human orthologs of zebrafish genes were identified using the R package biomaRt (*84*) based on zebrafish genome assembly GRCz11 and human genome assembly GRCh38.

### Gene set enrichment analysis

GSEA analyses for DisGeNET, GLAD4U, GO, KEGG, and REACTOME libraries were performed using the web-based gene set analysis toolkit (*83*) (WebGestalt; www.webgestalt.org/). GSEA analysis for the independent disease gene sets was performed using the GSEA software (*85*).

### Upstream enrichment signature and UES-blast

Upstream enrichment signature (UES) and UES-blast analyses were conducted in R using custom scripts. For each test gene set, we first run an ORA to find significantly enriched upstream regulators using the ChIP-X library (ENCODE_and_ChEA_Consensus_TFs_from_ChIP-X). As suggested by its full name, this library contains gene sets acquired by analyzing ChIP-X data in the ENCODE and ChEA databases. We then calculate a significance score for each significantly enriched (adjusted *P* < 0.05) upstream regulator as follows: significance score = −log(adjusted *P* value). Significance scores for all significantly enriched upstream regulators of a given gene set are normalized to between 0 and 1. All nonsignificant upstream regulators are assigned a significance score of 0. With a score assigned to each upstream regulator, we created a signature for each gene set, which we named UES.

We constructed a reference dataset containing >1700 UESs from analyzing published disease risk genes including DisGeNET and GLAD4U and the RNA-seq gene set library RNAseqGEO. To avoid variations caused by gene sets containing too few genes, only gene sets containing ≥500 genes from the DisGeNET library and ≥250 genes from the GLAD4U library were analyzed. We then conduct an unbiased search using a custom-written blast-style querying algorithm (UES-blast) to find gene sets with the lowest blast scores (highest similarity rankings) to a query gene set, e.g., SFARI genes. UES-blast was conducted by first calculating the Euclidean distances between test gene sets and the target gene set (e.g., SFARI genes) and then ranking test gene sets on the basis of their distances to the target gene set.

### Histone modification enrichment analysis and H3K27me3 score

We assessed the enrichment of histone marks in the promoter regions of genes in each test gene set using ORA . For each given histone mark, the curated ENCODE_HM and Epigenomics_Roadmap_HM libraries often contain a number of query gene sets each listing genes that are targeted by this histone mark in their promoter regions in a specific cell or tissue type. For example, a query gene set named “H3K27me3 cerebellum mm9” contains all genes that are marked by H3K27me3 in the mouse cerebellum, based on analyzing published ChIP-X data. In our analysis, we run ORA for each test gene set against all query gene sets in the two libraries to identify those that are enriched in our test gene set. We then count the number of significantly enriched query gene sets for each histone mark, such as the data shown in Fig. 6E.

To specifically quantify the enrichment of H3K27me3 in each test gene set, we calculated a H3K27me3 score as follows: H3K27me3 score = the ratio of significant H3K27me3 gene sets over all H3K27me3 gene sets in the library × the ratio of significant H3K27me3 gene sets over all significant histone mark gene sets = (the number of significant H3K27me3 gene sets/the number of all H3K27me3 gene sets) × (the number of significant H3K27me3 gene sets/the number of all significant histone mark gene sets). As an example, if a test gene set is significantly enriched for 60 H3K27me3 gene sets of a total number of 100 H3K27me3 gene sets in the library and, at the same time, also significantly enriched for another 10 assorted histone mark gene sets, then its H3K27me3 score is calculated as (60/100) × [60/(60 + 10)] = 0.6 × 6/7 = 0.51. For each test gene set, its final H3K27me3 score was acquired by first calculating two separate H3K27me3 scores using the ENCODE_HM and Epigenomics_Roadmap_HM libraries, respectively, and then combining these two scores by summation. All analyses were conducted in R using custom scripts.

### ChIP-seq data analyses

Genome-wide TOP2A binding data were acquired from GEO accession number GSE79593 (*50*); we used the DMSO-treated sample in this dataset for analyses. EZH2, SUZ12, and H3K27me3 ChIP-seq data were acquired from GEO accession numbers GSM1003576, GSM1003545, and GSM733658, respectively. To enable meaningful comparison with TOP2A binding peaks, all ChIP-seq data were acquired from the same cell line of K562 cells. Peaks from EZH2, SUZ12, and H3K27me3 that overlap with TOP2A peaks were identified using the R package GenomicRanges (*86*). Genes that are targeted by the original peaks or the overlapping peaks at their promoter or gene body regions were mapped using the R package bumphunter (*87*) and exported for subsequent analyses. Venn diagram for peak overlaps was drawn using the R package ChIPseeker (*88*).

### PPI analysis

PPI analysis, *k*-means clustering, and functional enrichment analysis of the clusters were conducted using STRING (*89*) (https://string-db.org/).

### Chromatin immunoprecipitation

Larvae (3 dpf) treated by Top2 inhibitors and rescue agent were sedated by tricaine (200 μg/ml; Western Chemical), collected into a 1.5-ml microcentrifuge tube, and euthanized on ice for 5 min. Euthanized larvae were washed once with PBS and homogenized by an electric microtube homogenizer (KONTES) for 30 to 45 s on ice while submerged in 200 μl of 37% formaldehyde in PBS. We add another 800 μl of 37% formaldehyde/PBS and cross-link at RT for 8 min on a rotator. Cross-linking was stopped by adding glycine to a final concentration of 0.125 M and rotating for another 5 min at RT. Samples were then kept on ice before moving on to the next step, spun at 3000*g* for 1 min at 4°C, the supernatant discarded, and washed twice with 1 ml of chilled PBS containing 1 mM phenylmethylsulfonyl fluoride (Cayman Chemical) and Halt Protease Inhibitor Cocktail (Thermo Fisher Scientific). After each wash, the samples were spun at 3000*g* for 1 min at 4°C, with the supernatant discarded. Samples could be stored at −80°C after this step. ChIP experiment was conducted by following the manufacturer’s protocol for the Pierce Magnetic ChIP kit (Thermo Fisher Scientific). Approximately 1 × 10^6^ cells were processed for each sample, and 4 μl of 1:10 diluted micrococcal nuclease was applied to each sample to fragment chromatin. Sonication was performed for three cycles with 20 s on and 20 s off at 40% amplitude. Each sample was divided into two equal parts and incubated with 1 μg of normal rabbit immunoglobulin G (IgG) antibody (Cell Signaling Technology, 2729S) as IgG control or with 1 μg of anti-H3K27me3 antibody (Cell Signaling Technology, C36B11).

### Zebrafish brain dissection and sample collection

Two-month-old male adult zebrafish were euthanized by submerging in icy water for 5 min. The head of the fish were cut off by scissors while submerged in ice-cold PBS, followed by eye and skull removal using two pairs of tweezers. Brain was carefully extracted from the skull and immediately placed in 200 μl of PBS on ice. Three brains were extracted for each sample. After dissection, brains were washed once in 1 ml of ice-cold PBS, flash-frozen in liquid N_2_, and stored at −80°C. RNA extraction was conducted using the QIAGEN RNeasy Lipid Tissue Mini Kit (QIAGEN). Reverse transcription was conducted using the QuantiTect Reverse Transcription Kit (QIAGEN) before qPCR.

### Quantitative PCR

We designed qPCR primers (table S1) and ChIP-qPCR primers (tables S2 and S3) using primer-Blast. ChIP-qPCR primers were designed to target the promoter regions (−1000 to +500 bp) of their zebrafish orthologs. qPCR was performed on a 7500 Fast Real-Time PCR System (Applied Biosystems) using PowerUp SYBR Green Master Mix (Applied Biosystems) by following the manufacturer’s protocol. For ChIP-qPCR data analysis, fold enrichment of H3K27me3 (compared to its corresponding IgG control) for each target gene was compared to the fold enrichment of the control gene eef1a1a to calculate an adjusted fold change (FC) as follows: adjusted FC = (target gene FC/eef1a1a FC)^treatment sample^/(target gene FC/eef1a1a FC)^DMSO control sample^.

## Notes

View/request a protocol for this paper from *Bio-protocol*.
