# Supplementary material for: Top2a promotes the development of social behavior via PRC2 and H3K27me3
Source: Sci Adv. 2022 Nov 23;8(47):eabm7069. doi: 10.1126/sciadv.abm7069 (PMC9683714; doi:10.1126/sciadv.abm7069)
Supplement: Supplementary file 1 — Figs. S1 to S14 Tables S1 to S3 [file sciadv.abm7069_sm.pdf]

Science Advances
AAAS

Supplementary Materials for

Top2a promotes the development of social behavior via PRC2 and H3K27me3

Yijie Geng *et al.*

Corresponding author: Randall T. Peterson, randall.peterson@pharm.utah.edu

Sci. Adv. **8**, eabm7069 (2022)
DOI: 10.1126/sciadv.abm7069

The PDF file includes:

Figs. S1 to S14
Tables S1 to S3
Legend for table S4
Legend for movie S1
Legend for data S1

Other Supplementary Material for this manuscript includes the following:

Table S4
Movie S1
Data S1

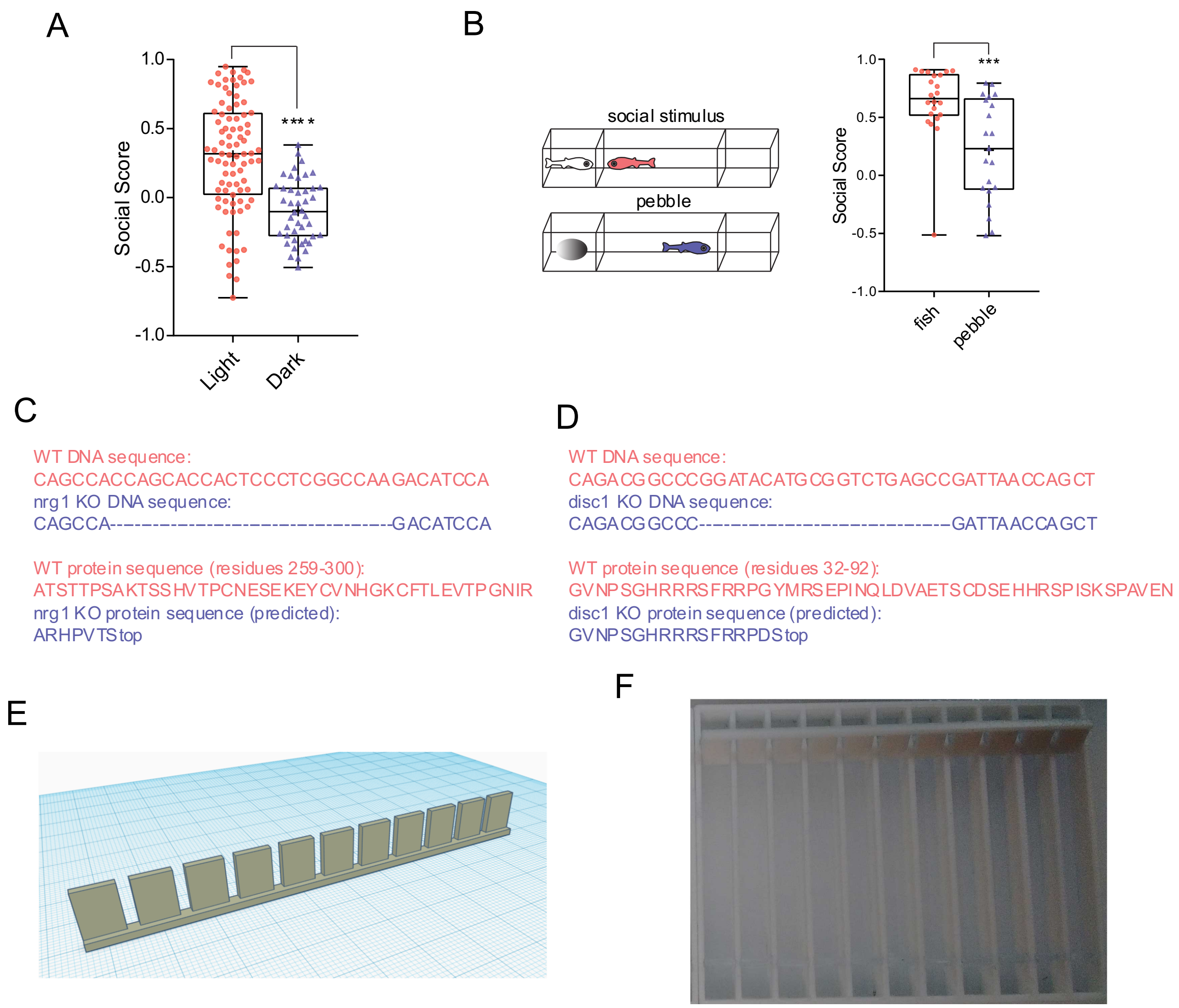

Figure S1. Setting up the Fishbook assay.
(A) Boxplot comparing social scores of WT fish in light (n = 87) or dark (n = 44). ****: p < 0.0001.
(B) Boxplot comparing social scores of WT fish in response to a social stimulus (fish; n = 22) or pebble (n = 21). ***: p < 0.001.
(C) DNA and protein sequences of WT and *nrg1* knockout fish.
(D) DNA and protein sequences of WT and *disc1* knockout fish.
(E) 3D printing design for the view-blocking comb.
(F) Image of a comb placed on a Fishbook test arena. The comb is not inserted in this image.

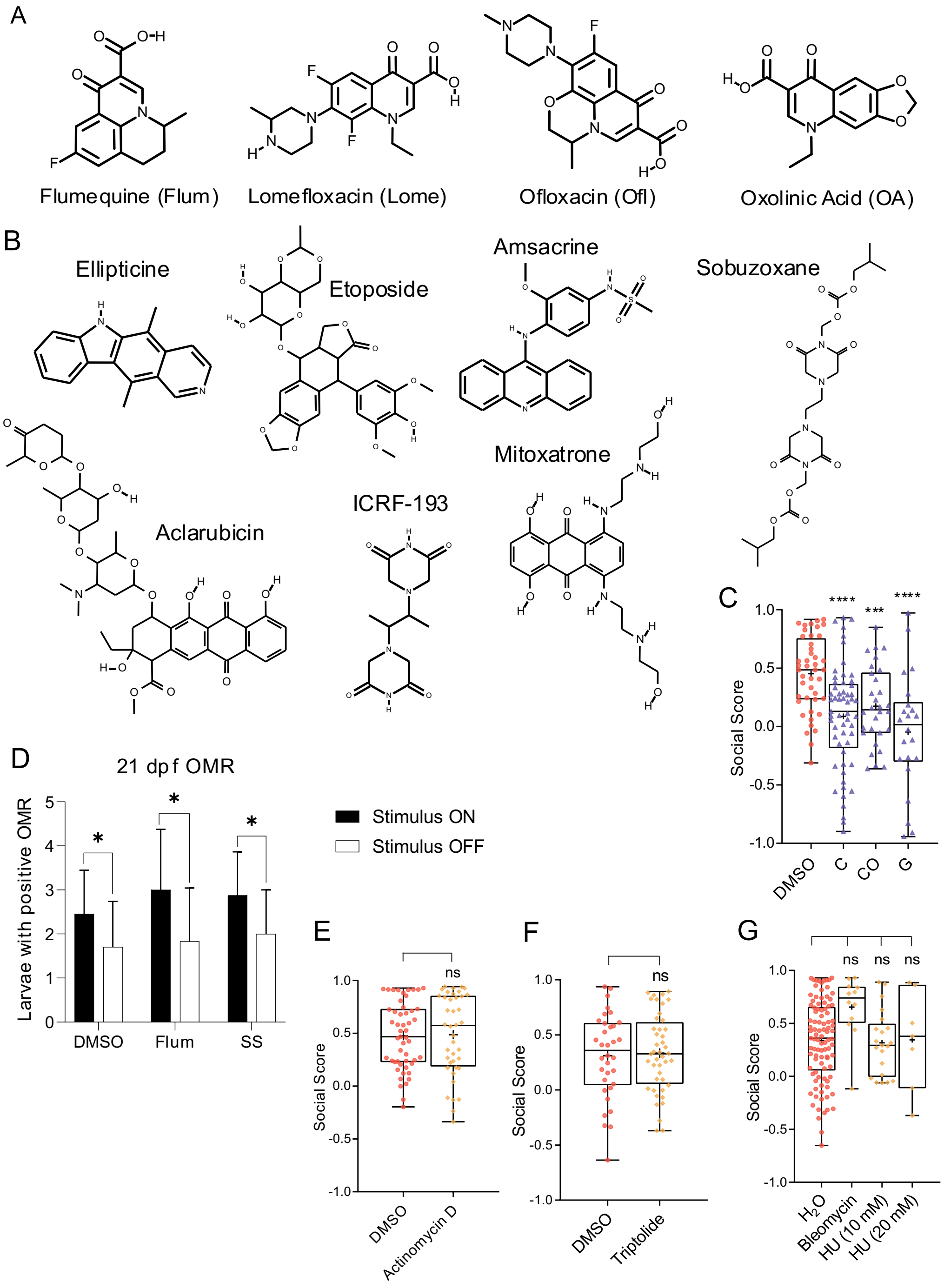

Figure S2. Validations and mechanism studies for Top2 inhibitor-induced social deficits.
(A) Chemical structures of fluoroquinolones.
(B) Chemical structures of eukaryotic Top2 inhibitors.
(C) Boxplot showing social scores of fish treated with DMSO (n=44), chlorpyrifos (C; 5 µM; n=57), chlorpyrifos oxon (CO; 1 µM; n=30), and genistein (G; 8 µM; n=22).
(D) Optomoter response (OMR) assay found significant differences in the number of larvae showing positive OMR (moving toward the same direction of the stimulus) between periods with or without stimulus (Stimulus ON/OFF) in 21 dpf larvae that received embryonic treatment of DMSO (n=12), 15 µM flumequine (Flum; n=6), and 100 µM sodium salicylate (SS; n=8),

indicating that vision is unimpaired in Top2 inhibitor treated fish.
(E) Boxplot comparing social scores of fish treated with DMSO (n = 51) or actinomycin D (20 μM; n = 40).
(F) Boxplot comparing social scores of fish treated with DMSO (n = 34) or triptolide (0.2 μM; n = 44).
(G) Boxplot comparing social scores of fish treated with DMSO (n = 97), bleomycin (1 μM; n = 12), or hydroxyurea (HU; 10 mM, n = 21; 20 mM, n = 7).
ns: not significant, ***: $p<0.001$, ****: $p<0.0001$.

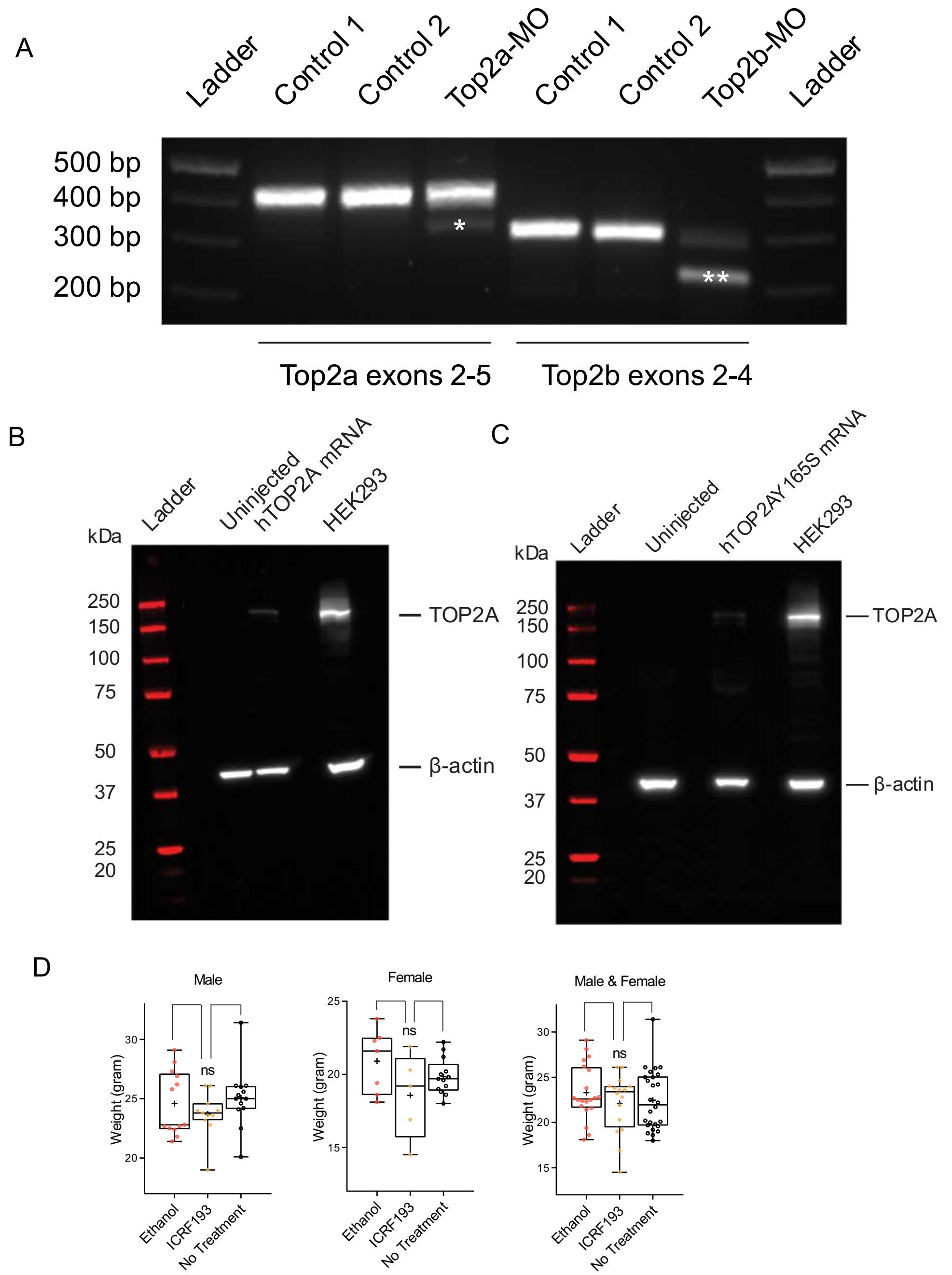

Figure S3. Validation of Top2a/b morpholinos and TOP2A overexpression.
(A) DNA gel image showing RT-PCR result of 1 dpf embryos injected with Top2a-MO (0.05 mM), Top2b-MO (0.05 mM), or un-injected (Controls 1 & 2). n = ~ 80 – 100 embryos for each condition. *: splice-blocked amplicon of Top2a. **: splice-blocked amplicon of Top2b.
(B, C) Western blot images showing protein overexpression following injection of 250 ng/µl *hTOP2A* (B) and *hTOP2AY165S* (C) mRNAs in zebrafish embryos. HEK293 cell lysate was used as positive controls. Uninjected zebrafish embryos were used as negative controls. β-actin was used as a loading control.
(D) Boxplot comparing the body weights of mice treated with ethanol (male: n = 13, female: n = 7), ICRF193 (male: n = 11, female: n = 5), and no-treatment control (male: n = 13, female: n = 13) at 2 months of age. ns: not significant.

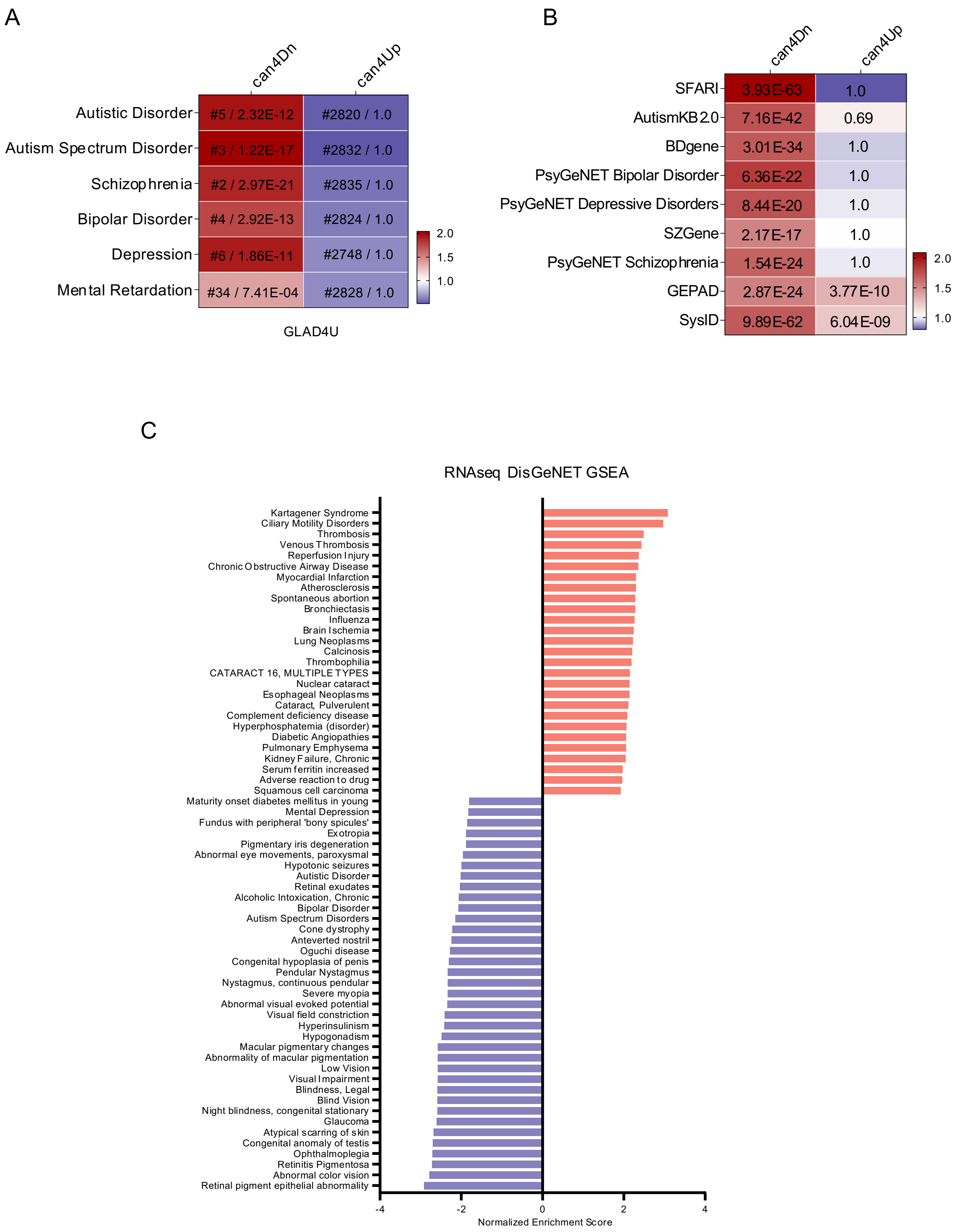

Figure S4. Downregulated genes in the can4 mutant are enriched for autism genes and autism comorbidity risk genes – ORA and GSEA in DisGeNET library.

(A) can4Dn but not can4Up is selectively enriched for autism and its comorbidities risk gene sets

from the GLAD4U library. For each cell, color represents odds ratio, and numbers represent ranking out of 3071 diseases in GLAD4U library (before slash) and adjusted *p*-value (after slash).

(B) Heatmap showing ORA analysis comparing can4Dn and can4Up using several independent disease risk gene sets related to autism and its comorbid disorders. Color in each cell represents odds ratio values. Number in each cell represents adjusted *p*-value.

(C) Bar chart showing normalized enrichment scores from GSEA analysis for RNA-seq data using the DisGeNET library.

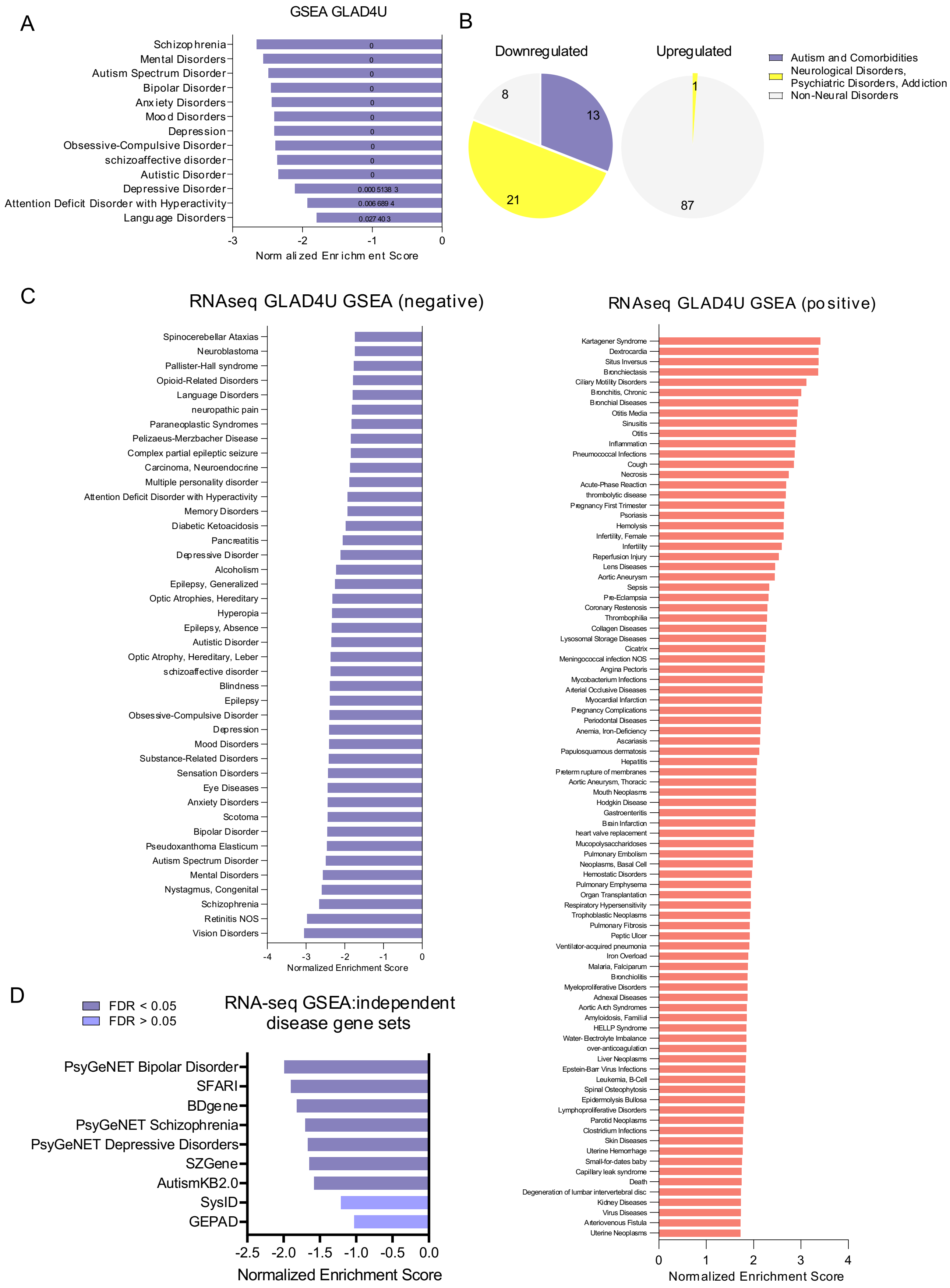
A
GSEA GLAD4U
Schizophrenia
Mental Disorders
Autism Spectrum Disorder
Bipolar Disorder
Anxiety Disorders
Mood Disorders
Depression
Obsessive-Compulsive Disorder
schizoaffective disorder
Autistic Disorder
Depressive Disorder
Attention Deficit Disorder with Hyperactivity
Language Disorders
0
0
0
0
0
0
0
0
0
0
0.000 5138 3
0.006 689 4
0.027 40 3
-3
-2
-1
0
Normalized Enrichment Score
B
Downregulated
Upregulated
8
13
21
1
87
Autism and Comorbidities
Neurological Disorders,
Psychiatric Disorders, Addiction
Non-Neural Disorders
C
RNAseq GLAD4U GSEA (negative)
Spinocerebellar Ataxias
Neuroblastoma
Pallister-Hall syndrome
Opioid-Related Disorders
Language Disorders
neuropathic pain
Paraneoplastic Syndromes
Pelizaeus-Merzbacher Disease
Complex partial epileptic seizure
Carcinoma, Neuroendocrine
Multiple personality disorder
Attention Deficit Disorder with Hyperactivity
Memory Disorders
Diabetic Ketoacidosis
Pancreatitis
Depressive Disorder
Alcoholism
Epilepsy, Generalized
Optic Atrophies, Hereditary
Hyperopia
Epilepsy, Absence
Autistic Disorder
Optic Atrophy, Hereditary, Leber
schizoaffective disorder
Blindness
Epilepsy
Obsessive-Compulsive Disorder
Depression
Mood Disorders
Substance-Related Disorders
Sensation Disorders
Eye Diseases
Anxiety Disorders
Scotoma
Bipolar Disorder
Pseudoxanthoma Elasticum
Autism Spectrum Disorder
Mental Disorders
Nystagmus, Congenital
Schizophrenia
Retinitis NOS
Vision Disorders
-4
-3
-2
-1
0
Normalized Enrichment Score
RNAseq GLAD4U GSEA (positive)
Kartagener Syndrome
Dextrocardia
Situs Inversus
Bronchiectasis
Ciliary Motility Disorders
Bronchitis, Chronic
Bronchial Diseases
Otitis Media
Sinusitis
Otitis
Inflammation
Pneumococcal Infections
Cough
Necrosis
Acute-Phase Reaction
thrombolytic disease
Pregnancy First Trimester
Psoriasis
Hemolysis
Infertility, Female
Infertility
Reperfusion Injury
Lens Diseases
Aortic Aneurysm
Sepsis
Pre-Eclampsia
Coronary Restenosis
Thrombophilia
Collagen Diseases
Lysosomal Storage Diseases
Cicatrix
Meningococcal infection NOS
Angina Pectoris
Mycobacterium Infections
Arterial Occlusive Diseases
Myocardial Infarction
Pregnancy Complications
Periodontal Diseases
Anemia, Iron-Deficiency
Ascariasis
Papulosquamous dermatosis
Hepatitis
Preterm rupture of membranes
Aortic Aneurysm, Thoracic
Mouth Neoplasms
Hodgkin Disease
Gastroenteritis
Brain Infarction
heart valve replacement
Mucopolysaccharidoses
Pulmonary Embolism
Neoplasms, Basal Cell
Hemostatic Disorders
Pulmonary Emphysema
Organ Transplantation
Respiratory Hypersensitivity
Trophoblastic Neoplasms
Pulmonary Fibrosis
Peptic Ulcer
Ventilator-acquired pneumonia
Iron Overload
Malaria, Falciparum
Bronchiolitis
Myeloproliferative Disorders
Adnexal Diseases
Aortic Arch Syndromes
Amyloidosis, Familial
HELLP Syndrome
Water-Electrolyte Imbalance
over-anticoagulation
Liver Neoplasms
Epstein-Barr Virus Infections
Leukemia, B-Cell
Spinal Osteophytosis
Epidermolysis Bullosa
Lymphoproliferative Disorders
Parotid Neoplasms
Clostridium Infections
Skin Diseases
Uterine Hemorrhage
Small-for-dates baby
Capillary leak syndrome
Death
Degeneration of lumbar intervertebral disc
Kidney Diseases
Virus Diseases
Arteriovenous Fistula
Uterine Neoplasms
0
1
2
3
4
Normalized Enrichment Score
D
FDR < 0.05
FDR > 0.05
RNA-seq GSEA:independent disease gene sets
PsyGeNET Bipolar Disorder
SFARI
BDgene
PsyGeNET Schizophrenia
PsyGeNET Depressive Disorders
SZGene
AutismKB2.0
SysID
GEPAD
-2.5
-2.0
-1.5
-1.0
-0.5
0.0
Normalized Enrichment Score

Figure S5. Downregulated genes in the can4 mutant are enriched for autism genes and autism comorbidity risk genes – GSEA in GLAD4U library.
(A, B) GSEA analysis of RNA-seq data using the GLAD4U library shows enrichment for autism and its comorbidities risk genes (A & B) and neurological conditions risk genes (B) in downregulated but not upregulated genes. Value inside each bar represents FDR (A). Significance: FDR<5%.
(C) Bar charts showing normalized enrichment scores (NESs) from GSEA analysis for RNA-seq data using the GLAD4U library. Left: hits with positive NESs; right: hits with negative NESs.
(D) Bar chart showing normalized enrichment scores from GSEA analysis for RNA-seq data using several independent disease gene sets related to autism and its comorbid disorders. Significance: FDR < 5%.

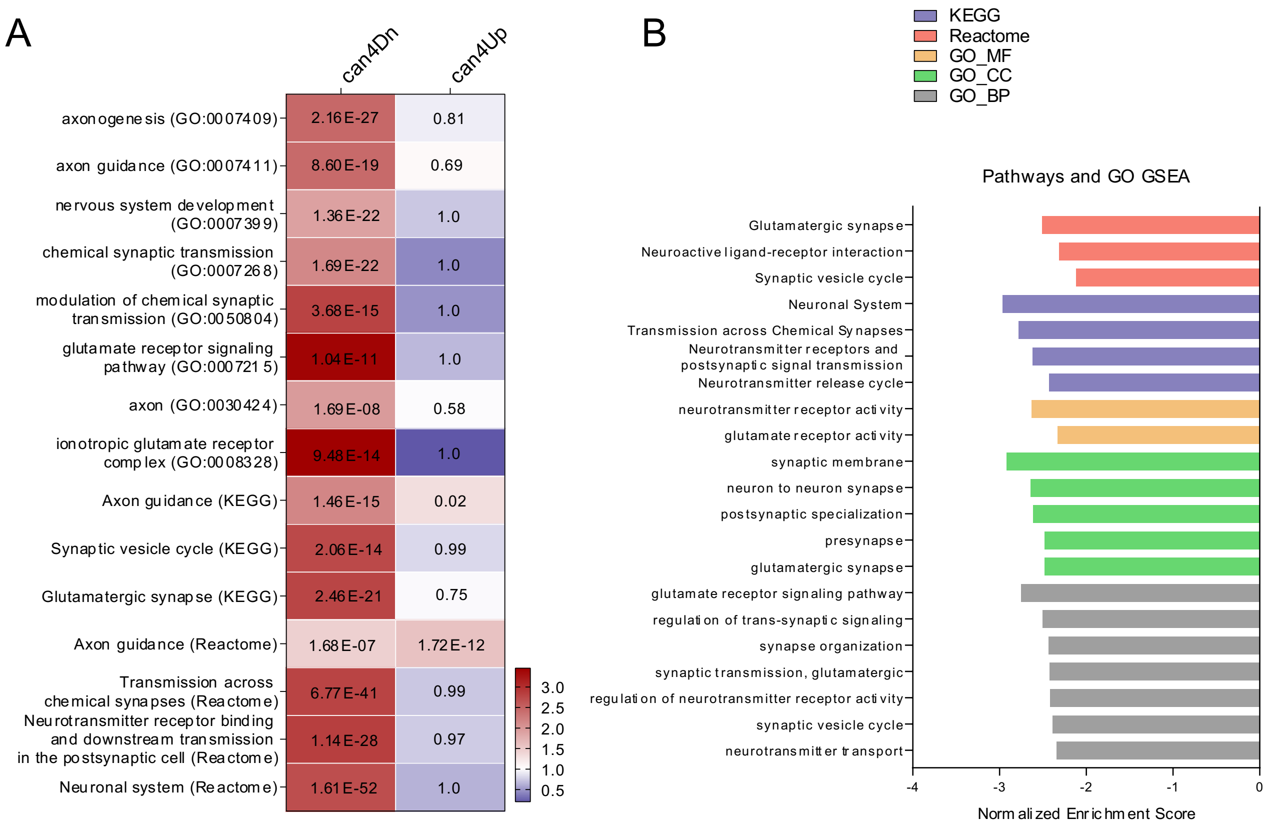

Figure S6. can4Dn but not can4Up genes are enriched for autism related biological pathways.

(A) Heatmap showing ORA analysis comparing can4Dn and can4Up using KEGG, REACTOME, and GO libraries. Color in each cell represents odds ratio.

(B) Bar chart showing GSEA analysis of KEGG, Reactome, and GO libraries using the RNA-seq data.

A

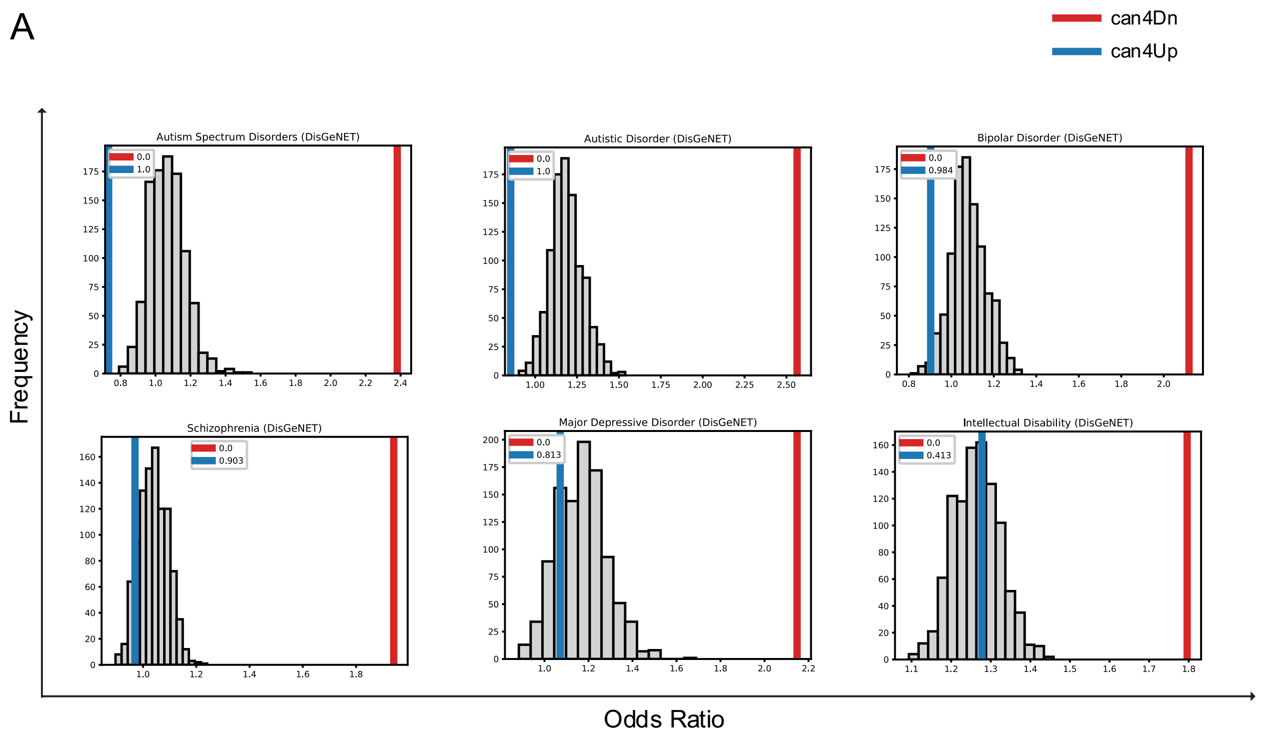

B

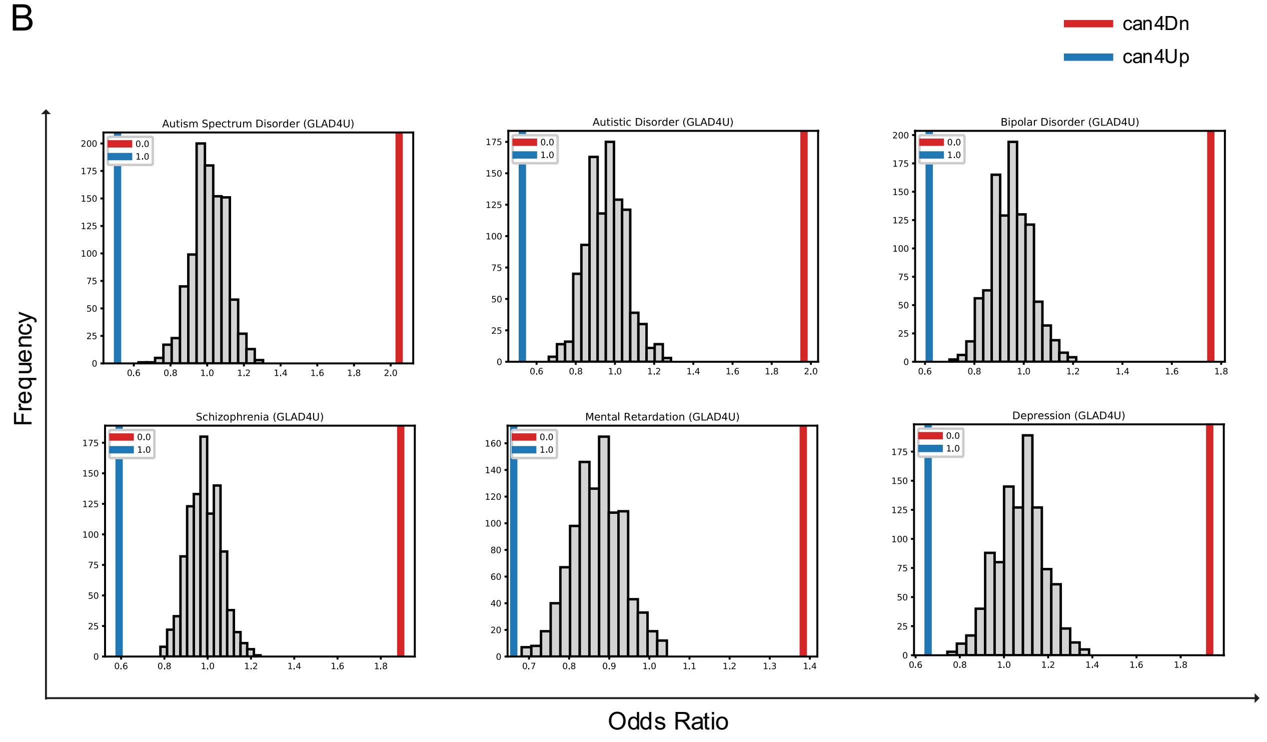

Figure S7. can4Dn but not can4Up gives significantly higher odds ratios compared to the null distribution of randomly selected zebrafish genes – testing DisGeNET and GLAD4U libraries.

(A) Histogram showing the null distribution of odds ratios generated by 1000 permutations by randomly selecting 5000 genes out of all human orthologs of zebrafish genes and conduct ORA

analysis using selected gene sets from the DisGeNET library. The odds ratios for can4Dn (red) and can4Up (blue) are marked by colored vertical lines, and their respective *p*-values are shown as numbers in legends. A *p*-value <0.008 was considered significant to correct for multiple comparisons.

(B) Histogram showing the null distribution of odds ratios generated by 1000 permutations by randomly selecting 5000 genes out of all human orthologs of zebrafish genes and conduct ORA analysis using selected gene sets from the GLAD4U library. The odds ratios for can4Dn (red) and can4Up (blue) are marked by colored vertical lines, and their respective *p*-values are shown as numbers in legends. A *p*-value <0.008 was considered significant to correct for multiple comparisons.

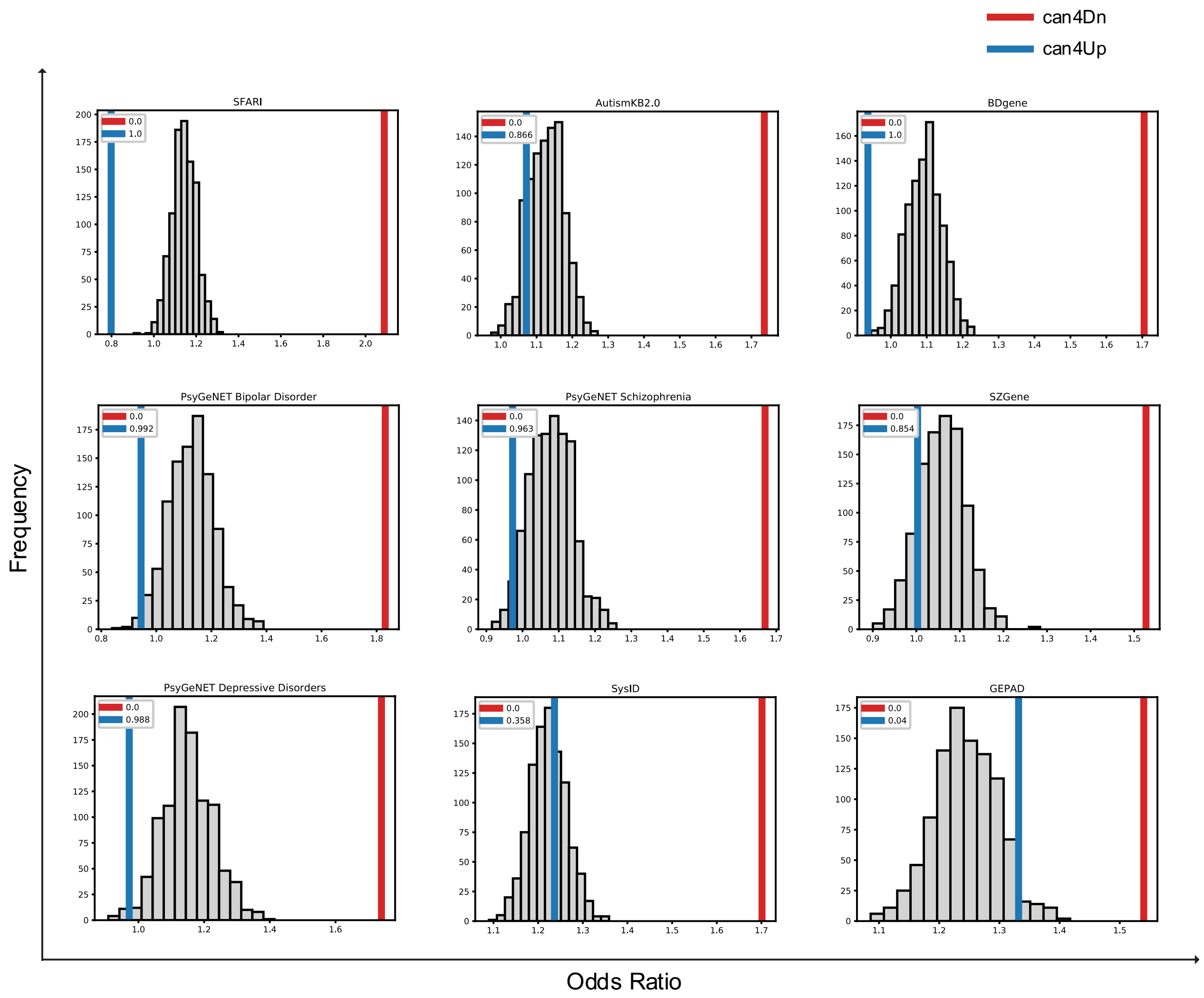

Figure S8. can4Dn but not can4Up gives significantly higher odds ratios compared to the null distribution of randomly selected zebrafish genes – testing independent disease gene sets related to autism and its comorbid conditions.
Histogram showing the null distribution of odds ratios generated by 1000 permutations by randomly selecting 5000 genes out of all human orthologs of zebrafish genes and conduct ORA analysis using selected gene sets from several independent disease gene sets related to autism and its comorbid conditions. The odds ratios for can4Dn (red) and can4Up (blue) are marked by colored vertical lines, and their respective *p*-values are shown as numbers in legends. A *p*-value <0.005 was considered significant to correct for multiple comparisons.

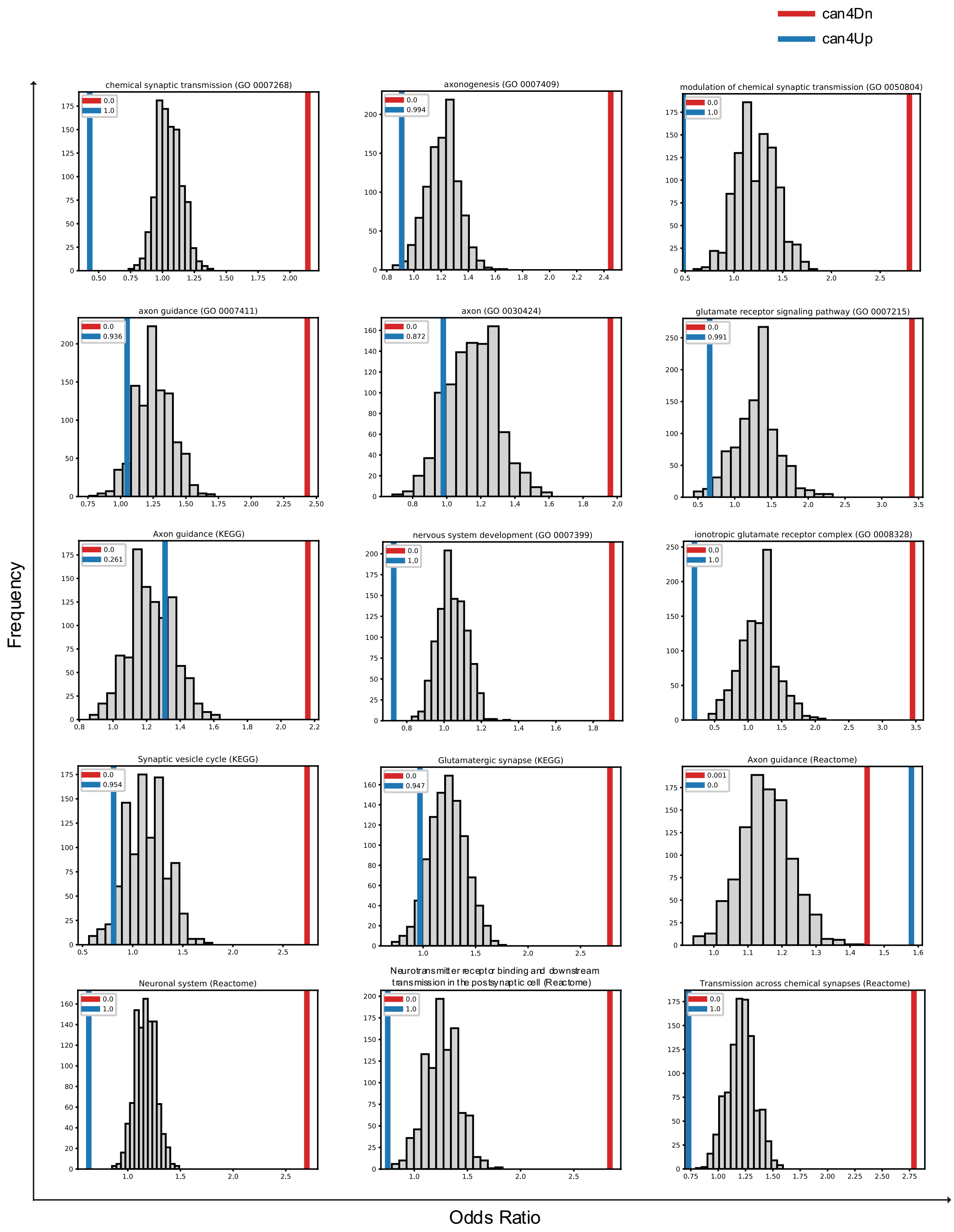

Figure S9. can4Dn but not can4Up gives significantly higher odds ratios compared to the null distribution of randomly selected zebrafish genes – testing KEGG, Reactome, and GO

libraries.

Histogram showing the null distribution of odds ratios generated by 1000 permutations by randomly selecting 5000 genes out of all human orthologs of zebrafish genes and conduct ORA analysis using selected gene sets from KEGG, Reactome, and GO libraries. The odds ratios for can4Dn (red) and can4Up (blue) are marked by colored vertical lines, and their respective *p*-values are shown as numbers in legends. A *p*-value <0.003 was considered significant to correct for multiple comparisons.

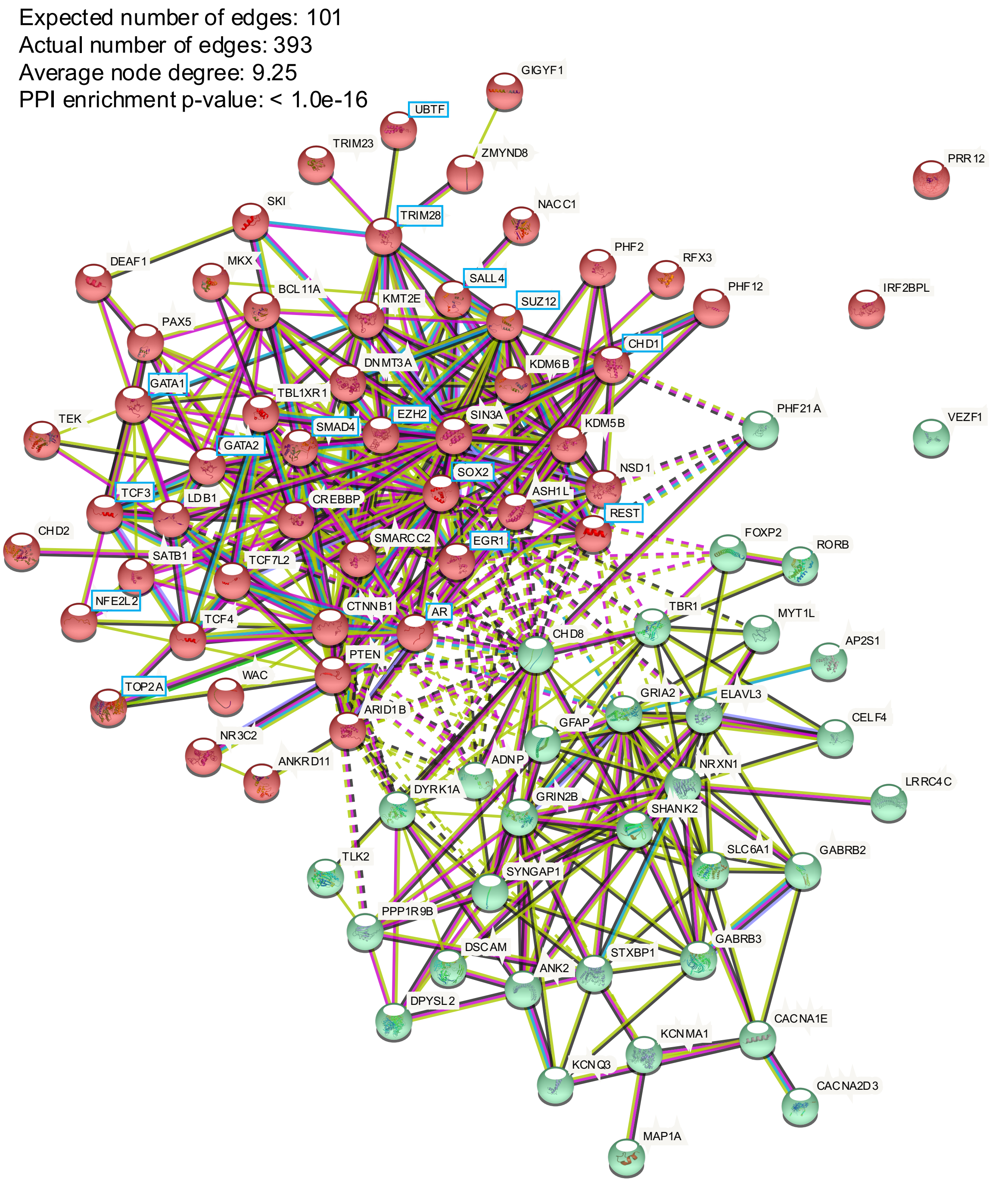

Functional enrichments			
GO-term	Description	Strength of Enrichment	FDR
GO:0035098	ESC/E(Z) complex	1.84	0.023
GO:0032452	Histone demethylase activity	1.62	0.0048
GO:0001102	RNA polymerase II activating transcription factor binding	1.55	4.78E-05
GO:0097107	Postsynaptic density assembly	2.3	0.012
GO:1904315	Transmitter-gated ion channel activity involved in regulation of postsynaptic membrane potential	1.65	0.0016

Figure S10. The 15 common upstream regulators of SFARI and can4Dn genes are functionally integrated with 70 Satterstrom-can4Dn genes at the protein level. Protein-protein interaction (PPI) network of Top2a, the 15 common upstream regulators of

SFARI and can4Dn genes, and 70 Satterstrom-can4Dn genes. The network is divided into two clusters by kmeans clustering, as shown by the nodes colored red and green. The blue rectangles label Top2a and the 15 common upstream regulators of SFARI and can4Dn genes: all these factors are assigned to the red cluster. Table shows the functional enrichments of the two clusters, with the red cluster enriched in genes associated with chromatin modification and transcriptional regulation while the green cluster is enriched in genes associated with synaptic activity regulation.

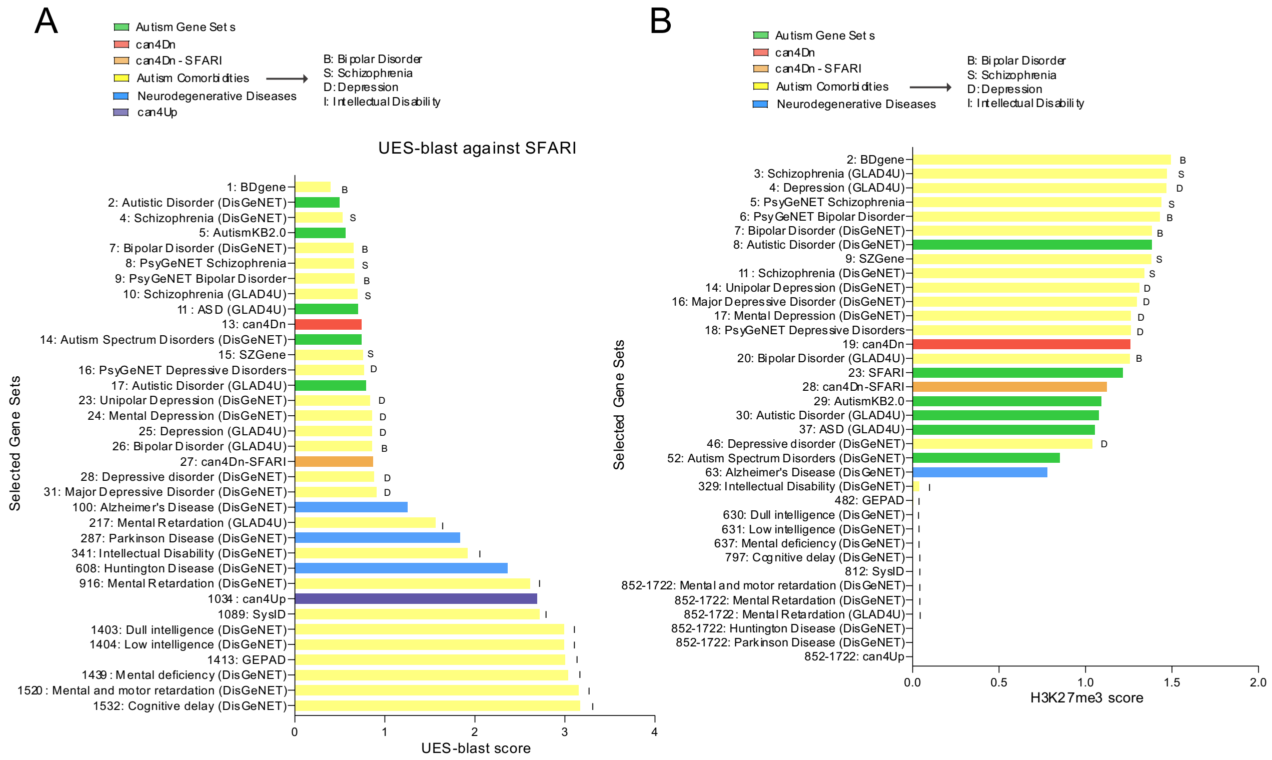

Figure S11. Detailed looks at UES-blast and H3K27me3 score rankings.

(A) A detailed look at UES-blast rankings of the key gene sets in Figure 6C. Bar chart shows the UES-blast scores of autism risk gene sets (green), can4Dn (red), can4Dn-SFARI (orange), autism comorbid conditions risk gene sets (yellow; D: depression, B: dipolar disorder, S: schizophrenia, I: intellectual disability), can4Up (dark blue), and neurodegenerative disorders risk gene sets (blue). Numbers in the x-axis labels before gene set names represent the UES-blast rankings of each labeled gene set.

(B) A detailed look at H3K27me3 score rankings of the key gene sets in Figure 6F. Bar chart shows the H3K27me3 scores of autism risk gene sets (green), can4Dn (red), can4Dn -SFARI (orange), autism comorbid conditions risk gene sets (yellow; D: depression, B: dipolar disorder, S: schizophrenia, I: intellectual disability), can4Up (dark blue), and neurodegenerative disorders risk gene sets (blue). Numbers in the x-axis labels before gene set names represent the H3K27me3 score rankings of each labeled gene set.

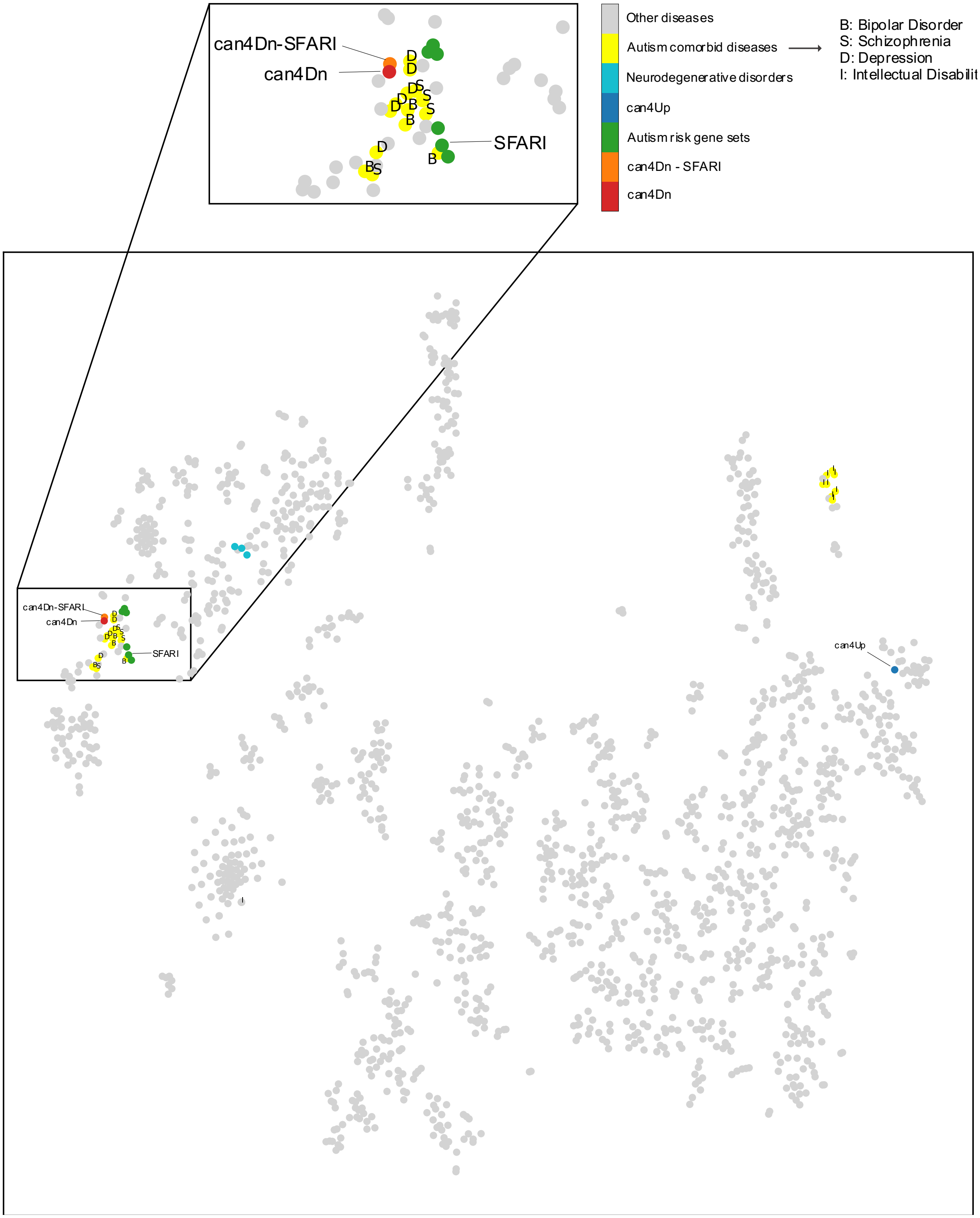

Figure S12. The upstream enrichment signatures (UESs) of can4Dn, SFARI, and other autism risk gene sets tightly cluster together compared to the reference dataset.
tSNE clustering of can4Dn, can4Dn-SFARI, autism risk gene sets, neurodegenerative disorders risk gene sets, and all control gene sets from the reference dataset. Letters label autism

comorbidities risk gene sets, I: intellectual disability; D: depression; S: schizophrenia; B: bipolar disorder.

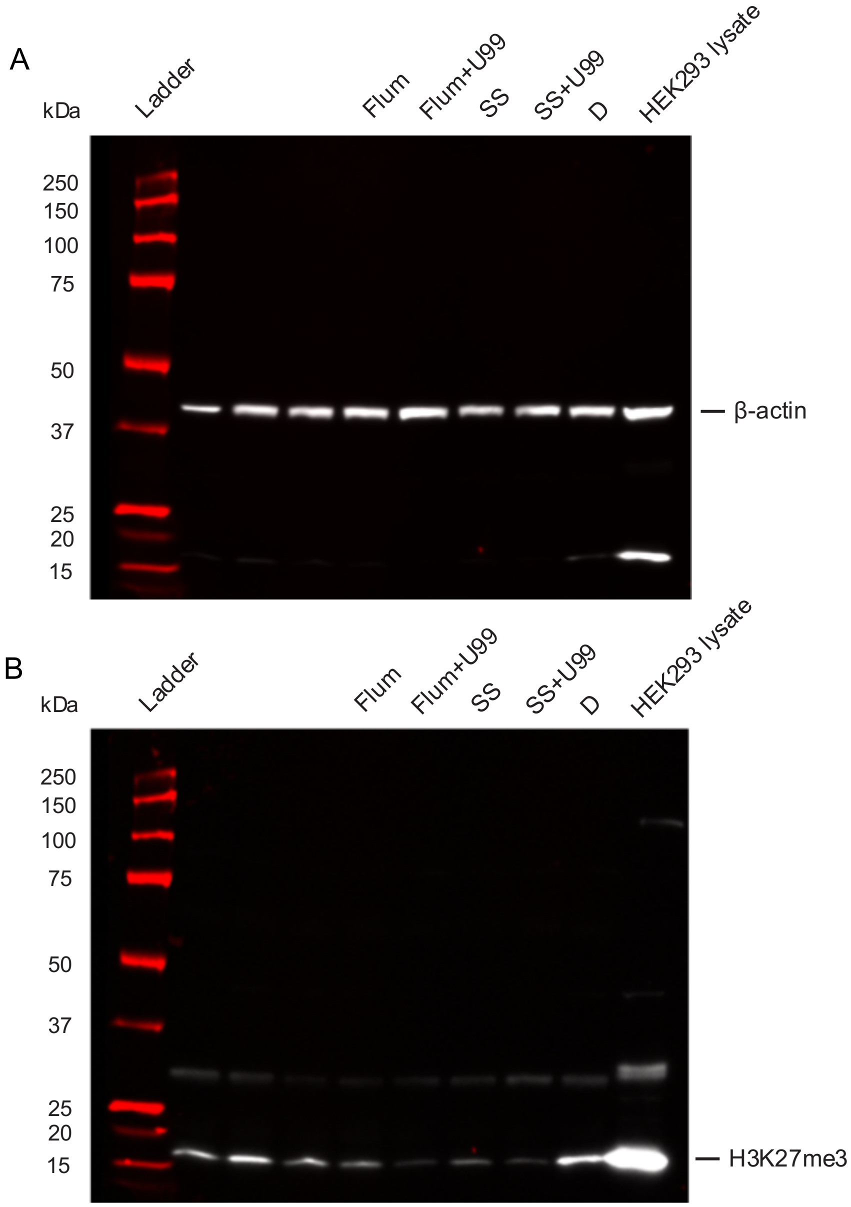

Figure S13. The raw Western blot images corresponding to data in Figures 8C.
Raw Western blot images of β-actin (A) and H3K27me3 (B) blotting. Cropped images are shown in Figure 8C.

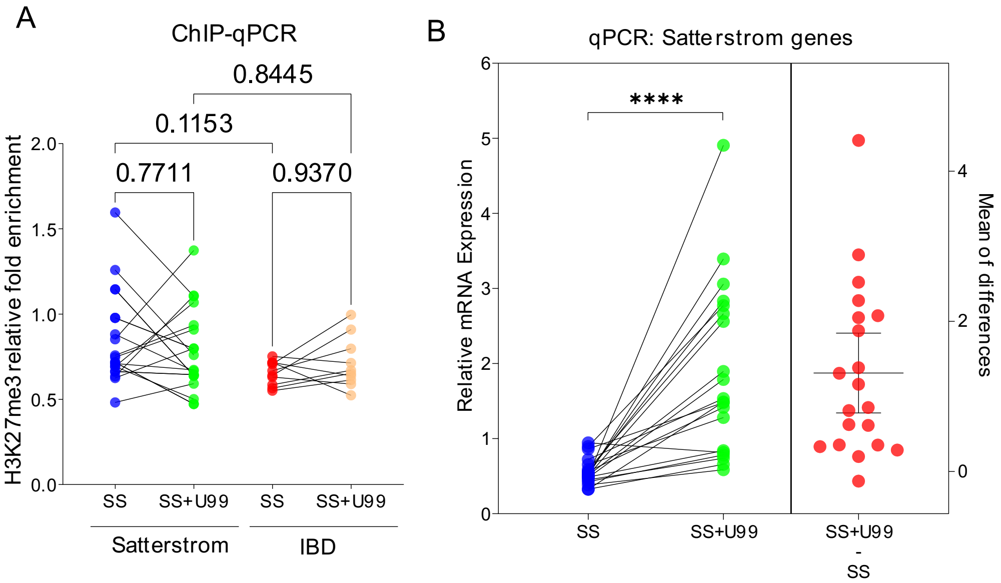

Figure S14. UNC1999 rescues changes in H3K27me3 and gene expression induced by Top2a inhibition.

(A) ChIP-qPCR shows differences in the relative fold enrichment of H3K27me3 (compared to the H3K27me3 level in the promoter region of *eef1a1a* gene) in zebrafish orthologs of 13 top ranking Satterstrom genes (Satterstrom) versus IBD risk genes (IBD) in 3 dpf larvae treated with 100 µM sodium salicylate (SS). The promoter regions of Satterstrom genes (blue) have a higher relative level of H3K27me3 compared to the IBD genes (red). Co-treatment of 10 µM UNC1999 (U99) reduced the relative level of H3K27me3 in the Satterstrom genes (green) and increased that in the IBD genes (brown), therefore rebalancing the level of H3K27me3 between Satterstrom and IBD genes. Each dot represents the relative fold enrichment of H3K27me3 for a single gene. Numbers indicate adjusted *p*-values using ordinary one-way ANOVA and Tukey's multiple comparisons test.

(B) qPCR shows downregulation of the zebrafish orthologs of the 13 top ranking Satterstrom genes in brain samples collected from adult zebrafish that received embryonic treatment of 100 µM sodium salicylate (SS; using the same data shown in Figure 5E) compared to DMSO control. Co-treatment of 10 µM UNC1999 (U99) at the embryonic stage rescued gene expression. Differences in relative mRNA expression levels between these two samples (SS+U99 - SS) are shown as red dots, with the horizontal bars representing mean and standard deviation of the differences. Significance is calculated using paired t test. ****: $p<0.0001$.

Table S1. qPCR primers for Satterstrom genes.

Human Gene	Zebrafish Ortholog	Forward Primer	Reverse Primer
ADNP	adnpa	GGGAATTCCATGCGAGACCG	ACCGGGTGAGGTTATTCACTGG
	adnpb	TCTTTCCCGGCAGACAGGCA	TGCTGAGGTTGTTCACTGGGA
ARID1B	arid1b	ACGCCTCTCATCAGAGCAGAC	TAGGCTTCACTCTCGTGCCG
CHD8	chd8	CCTCGGTTACTCACGGGTGG	ACTCCCTTCACCGTTTGGCA
DYRK1A	dyrk1aa	GATCGAAGTGCGACTGCTGG	TTACGCGTCAGGTTCAGGGA
	dyrk1ab	ACGCTGTTGTACCTGTCAGC	CGAGGGTTTGCATGCTGAGG
FOXP1	foxp1a	CATTGCCGTCAGTCACAACCC	GGGGACTTCTCACCACTTGCT
FOXP2	foxp1b	TAGTGACAGCAGCCCTGGAC	GGGAGAAACCAGGCTTACAGCA
GRIN2B	grin2bb	GACTTCTCCCCTCCGTTCCG	TCAGGACCGCTGCATCGTAG
KMT5B	kmt5b	AGGCTTGAGCCAGAAGGAAA	TCAACAAGACGGCGACAGGT
POGZ	pogza	CTGCAATGTCACGTCGAGGC	CCAGGCTTGTGCACGTGTTT
	pogzb	ATCATGTTCCTGGAAGGGCTCC	GGCAGAACTCAATATCTGTCTCCA
PTEN	ptena	CCCAGGGCTTCAAGAAAGGCA	GTGTGGGCTGGATTTGACGTG
	ptenb	AGACGGGTTCGACTTGGACT	ACACCCTCCAGTCTTTCAGCA
SHANK3	shank3a	TCCGGTTGTGCTTTTTGCGA	GCTTATCCATCACCGCCAGC
	shank3b	TTCACAGACCAGAGAAGGACC	GGCAGGTGGACTGATAGGCA
SLC6A1	slc6a1a	GCAGTTGCCCGTTTCACCAC	CCAGAGCAGTGATGAAGCCCT
	slc6a1b	GCATCTGGAAGGGCGTAGGA	TTGCTGAACTCAGGCGTCAC
SYNGAP1	syngap1a	TCCGTGCGCTGTACGAGTCT	CCTTCAGCTCACGGGGAAAC
	syngap1b	GGGCCTTCGAGGGCTACATT	GGCATCCTTTGACACAATAAACCA

Table S2. ChIP-qPCR primers for Satterstrom genes.

Human Gene	Zebrafish Ortholog	Forward Primer	Reverse Primer
ADNP	adnpa	CCGGTGGATTTCGGCAACTC	CCCGTCTAAACCGCGCAAAT
	adnpb	GGAAAACAGTATGCCACATCCCA	TCCTGACTGTGAGGGAGGAGA
ARID1B	arid1b	AAGTCTCCGCTCGCCTCAGA	TTGAGGATGGAGCGTTCGCC
CHD8	chd8	CCCTCTGCCAACCGACCAAT	ACAGGAAACGGCCAGCGTAG
DYRK1A	dyrk1aa	ACATCTGCAGAGACTCGGAGC	GTTCAGTGACCTCAGCGTGC
	dyrk1ab	CATCCGGGTACTAGGCTGCT	TTATTCCGGCGGGAAGCGAA
FOXP1	foxp1a	AGCGTACACATGACGGTTCG	TCAGCTCTCCCAGGACCACT
FOXP2	foxp1b	AGATGCGTTACTGGCCCTGG	AGGAGATCTCACCGCGACAC
GRIN2B	grin2bb	GCAGTAATCCTGCAGAGGGACC	GGACAGAGCGCTGTGGAGTA
KMT5B	kmt5b	TAGCGCTCGATCACCATCCA	AGACGACAGGACAACCGAGC
POGZ	pogza	TACCAGCGCGAGCAAAGAGG	GCTTTAACTCTTCGCGGGCTT
	pogzb	CCGGCAGGCTTTGTGTTCTTT	CTGTCCTCCGCGCACATAAC
PTEN	ptena	GAGGAGAGATGAGTCCCGCC	TGCGTGTTAGATGTGCGACG
	ptenb	TGCGCTCGCAGTAACCCTAA	TTGTTGAGCTGACATGGCGT
SHANK3	shank3a	AAGACCACTGAAGGGGCGATTA	CTGCTTATTTACTGTGCTCTGTCT
	shank3b	AGCATCAGGGTTGGGCTTCA	TCGCAGGCACTTTTCAGAGC
SLC6A1	slc6a1a	AGACAGTCGTTGCTCGGTGG	CCATGTAAAGCGGTCCTGCG
	slc6a1b	ACTGGACGCGGATTCTAGACG	AAAGTGCGGTGGTGCTGAAG
SYNGAP1	syngap1a	CGATCAGCTTACTCATTGGCCG	CGCCGATCACTTTACGCTCA
	syngap1b	GTGATGCGATACACGGGCTC	CGCTGGAGCGATATTCCCGT

Table S3. ChIP-qPCR primers for IBD genes.

Human Gene	Zebrafish Ortholog	Forward Primer	Reverse Primer
PTGS2	ptgs2a	TTTGCGCAAGACCTCCTCC TC	GTTACTCTGGTCGAGCCCGTT
	ptgs2b	CTGAGCAGGTAGCCTACGC C	TGAGGAGCCGATGACGCAAT
LY75	ly75	GGCGAGGCAACATCGGTTA AG	CATACGCGTTCACAGGCCG
PLA2R 1	pla2r1	CGCGACGCAACGGGAATC	CCGTCTGTTCTGACCGCGAC
NFKBI Z	nfkbiz	TGGTCTGAGAGCAGAAGTC GCC	ACACACGTCATTACGTCCAG GT
OSMR	osmr	TGCTCTGTAGATGGAGCTC TGC	GCGGTCCAGAAATCACTGGG T
PTK2B	ptk2ba	TCACACTTCAGGTCTGTCA AGC	GCACTTCCTTCTCAGTGTTTG AGTG
	ptk2bb	ACCACTACCACCACTGTTT ACCC	ACAGTGTCTCCTAAAGCAAG CAT
SH2B3	sh2b3	TGTTTGGCTTGCTGAGCTTA TG	AAGGTGGACTCTATGGGTGC TG
NFATC 1	nfatc1	TCTGCTGTTGCGCTTGAGA C	AAAGCCGGGAAACACCTCCG

Table S4.
A list of all genes included in the BDgene gene set.

Movies S1.
Video recording of a Fishbook test. Played at 4× the actual speed.

Data S1.
The can4Dn and can4Up gene lists.
